# Supply chain coordination strategy for NEVs based on supplier alliance under dual-credit policy

**DOI:** 10.1371/journal.pone.0257505

**Published:** 2021-10-01

**Authors:** Miaomiao Ma, Weidong Meng, Yuyu Li, Bo Huang

**Affiliations:** 1 School of Economics and Business Administration, Chongqing University, Chongqing, China; 2 School of Economics and Management, Chongqing Normal University, Chongqing, China; Sunway University, MALAYSIA

## Abstract

In this paper, we assume that the supply chain for new energy vehicles (NEVs) consists of a manufacturer and N parts suppliers, considering that the R&D investment of both manufacturer and suppliers will affect the market demand of NEVs and NEVs credit, we construct decentralized and centralized decision-making models under the dual-credit policy to study the R&D investment strategy of supply chain enterprises. Furthermore, considering that suppliers can form alliances, we establish bargaining game models under the conditions of the non-alliance and alliance of suppliers, and discuss the coordination strategy for the NEVs supply chain. It is found that, under the dual-credit policy, the higher the credit coefficient of technology improvement, the higher the transaction price of credits, and the higher the R&D investment of supply chain. Dual-credit policy can effectively encourage NEVs supply chain to increase R&D investment, improve NEV technology level, and improve the profit of supply chain. Under the dual-credit policy, the increment profit distribution strategy based on a bargaining game model can coordinate the NEVs supply chain. When suppliers separately negotiate with the manufacturer, bringing the negotiation sequence forward, the supplier can get more profits. However, as the manufacturer has the right to determine the negotiation sequence, the supplier can only get the profit of the last round of negotiation, and the manufacturer can get excess profit. Forming a suppliers alliance can solve this problem effectively, and increase the profit of all suppliers when the alliance`s negotiating power is improved to a certain threshold.

## Introduction

The development of NEVs is an important means for alleviating environmental pollution and energy shortages in China [1]. In order to realize the large-scale promotion of NEVs, improving the technical level is key [2]. In 2017, the Ministry of Industry and Information Technology and five other ministries and commissions jointly issued the “Parallel Management Measures for the Average Fuel Consumption of Passenger Vehicle Enterprises and Credits for New Energy Vehicles” (referred to as the " Dual-credit Policy ") [3], in order to improve the technical level of NEVs through market driving forces. Under the dual-credit policy, manufacturers can obtain a certain number of new energy credits for producing NEVs. The higher the technical level of NEVs, the higher the credits will be. New energy credits will become a tradable commercial resource and a new source of income for manufacturers [4], and thus manufacturers have the incentive to increase R&D investment and improve their technology level. However, the improvement of the technical level of NEVs largely depends on the improvement and breakthrough of the technical level of parts [5], and on the R&D investment of suppliers, which may not have the motivation to increase their R&D investment. Therefore, under the dual-credit policy, how to motivate suppliers to increase R&D investment to jointly improve the technical level of NEVs and achieve the coordination of the NEVs supply chain is crucial to the development of NEVs.

Since the implementation of the dual-credit policy, scholars have begun to study the impact of the dual-credit policy on the production and operation of enterprises. Tang et al. studied the optimal production strategy for a manufacturer producing NEVs and fuel vehicles under the dual effects of policy drivers and consumer preferences [6]. Zhang et al. studied the optimal production decisions and optimal social welfare of automobile enterprises under three different market structures: passenger vehicles complete market, NEVs manufacturers with market power and traditional fuel vehicles with market power [7]. Li et al. established a multi-period dynamic equilibrium model of the integral market, and compared the changes of the optimal output, credits and profit of the NEVs manufacturers under the conditions of decelerated growth, accelerated growth, continuous growth and an unchanged benchmark ratio [8]. Ou et al. established a new energy and oil consumption credits model to quantify and study the impact of a dual-credit policy on the corporate profits. However, the research of these scholars has mainly focused on the impact of the dual-credit policy on the production operation of a single enterprise [9]. Some scholars also studied the impact of the dual-credit policy on the production strategies of the two enterprises, for example, Cheng and Mu built a model based on the shareholding ratio and internal option agreement to study the joint production decision problem of two automobile manufacturers under three situations of credit equilibrium, credit surplus and credit shortage [10]. Lu and Yan established a three-stage game model for the duopoly of NEVs enterprises and found that the R&D cooperation between NEVs enterprises can improve the range capacity of NEVs, the total profit of enterprises and social welfare, which would be more favorable for the NEVs enterprises [11]. However, these scholars mainly studied the impact of a dual-credit policy on two horizontal enterprises. Some scholars have also begun to study the impact of a dual-credit policy on the production strategy of the NEVs supply chain. Zheng et al. established a three-stage game between NEVs manufacturers and suppliers, indicating that only the combination of an R&D subsidy policy and dual-credit policy can promote the technological innovation of enterprises [12]. Ma and Guo analyzed the optimal strategy combination of NEVs manufacturers and battery manufacturers in the cases of centralized decisions and independent decision, and the results showed that the R&D cooperation of the NEVs supply chain was better than the R&D competition [13]. Although the above research has achieved certain theoretical results, it is believed that the R&D cooperation between supply chain enterprises would be more favorable under the dual-credit policy. However, first, these studies are only limited to the cooperation between two enterprises, which is far from the reality. Secondly, these studies only consider the relationship between enterprises to be a complete cooperation; in fact, modern market competition features competition between the whole supply chain, including the upstream and downstream enterprises in the supply chain, and thus, the relationship is somewhere between cooperation and competition, with companies maximizing the profit of the supply chain while maximizing their own profit. Thirdly, these studies mainly use the master-slave game model, which assumes that the dominant party has the complete right to decide the cooperation strategy, without considering that a large amount of R&D cooperation is realized based on bargaining negotiations.

The most popular model used to analyze the negotiation in cooperation is the Nash Bargain Model proposed by Nash in 1953 [14]. For example, Escapa and Gutierrez studied the distribution of potential benefits of environmental cooperation between countries based on the bargaining game model [15]. Based on the bargaining game model, Xu et al. studied the profit distribution problem regarding the incremental profit generated by the joint operation of multi-agent reservoir groups [16]. Gong et al. designed a three-stage reverse supply chain system and established the Nash negotiation model to distribute the overall profit; it has found that the profit income of supply chain members was proportional to their status in the chain [17]. Li established the bargaining model of three-tier production planning and distribution planning in the collaborative planning system of a three-tier supply chain and realized the Pareto optimal solution [18]. Gong et al. used the Nash bargaining negotiation model and designed the benefit distribution method of a three-level supply chain system, on the base of cooperation satisfaction [19]. Marx et al. believed that bargaining power would affect the transaction conditions of negotiations between agents. When multiple parties with interrelated interests negotiate, the outcome of each negotiation depends on the bargaining power of each party [20]. Zhao et al. built a principal-agent model based on conversion cost and studied the internal mechanism of influencing the bargaining power of supply chain members [21]. Yang and Ou studied the effects of revenue sharing and negotiating power on producers’ carbon emission reduction decisions and members’ benefits in the context of government’s carbon tax and consumers’ preference for low-carbon products [22]. Zhang et al. found that the asymmetry of negotiation ability of supply chain members would affect suppliers’ input in quality improvement [23]. Therefore, when the supply chain makes decisions based on the bargaining model, the negotiating power will have an impact on the decision-making of all parties. There may be great differences in negotiating power among NEVs supply chain members, so it is necessary to study the impact of negotiating power on NEVs supply chain enterprises.

In addition, in the NEVs supply chain, manufacturers purchase auto parts from suppliers, and suppliers may form alliances to cooperate with manufacturers. Some research has been conducted on supplier alliances. For example, Nagarajan and Sošić considered a demand-determined supply chain composed of an assembler and multiple suppliers, where suppliers formed a dynamic alliance to compete with the assemblers. They studied the stability of the supply chain alliance under three different alliance modes [24]. On the basis of [24], Sošić further considered the stability of a supply chain alliance under three different alliance modes when the demand for finished products from the assemble to order supply chain was uncertain [25]. In contrast to the literature mentioned above, our study attempts to explain that during the negotiation process, suppliers form alliances in order to increase their own profits, to achieve the purpose of coordinating the NEVs supply chain.

## Problem description and model building

### Problem description

This paper assumes an NEVs supply chain composed of a manufacturer and N parts suppliers; the manufacturer purchases complementary parts from N parts suppliers for production, and the manufacturer sells the NEVs to the customer after the completion of production. The R&D investment of both manufacturers and suppliers will affect the market demand of NEVs. The member enterprises of the NEVs supply chain can make decentralized decisions; that is, the manufacturer and supplier decide the R&D investment and price through the Stackelberg game model with the aim of maximizing their own profits. Members can also perform centralized decision-making, aiming at maximizing supply chain profits, jointly decide R&D investment, and then decide parts prices and profit distribution through the bargaining game.

The NEVs supply chain makes decentralized decisions, and the decisions of the manufacturer and supplier are divided into two stages: in the first stage, manufacturer decides his own R&D investment and the price of NEVs according to the price of parts, the R&D investment of suppliers and the dual-credit policy. In the second stage, suppliers decide the R&D investment and price of parts.

The NEVs supply chain makes centralized decisions, and the cooperation process is as follows: first, suppliers decide whether to form an alliance to participate in the supply chain cooperation and make profit distribution rules. Then, the manufacturer and the suppliers make decisions with the aim of maximizing the total profit of the supply chain, including R&D investment, NEVs sales price, and the negotiation sequence in the case of non-aligned suppliers. Since all the suppliers have to negotiate with the manufacturer, the manufacturer has the right to determine the negotiation sequence. Next, if the supplier does not form an alliance, the manufacturer will negotiate with the supplier in the sequence of negotiations, determine the price of the supplier’s parts and the profit of each supplier, and sign a contract. If suppliers form an alliance to participate in supply chain cooperation, the manufacturer will first negotiate with the supplier alliance to determine the profit of the supplier alliance, then a profit distribution will be conducted within the supplier alliance according to the agreed rules to determine the profit and parts price of each supplier, and a contract will be signed with the manufacturer. Finally, the NEVs supply chain is committed to R&D investment, procurement and sales in accordance with contracts to achieve their respective profits.

Obviously, the profit of manufacturers and suppliers is affected by many factors, such as negotiating power, the negotiation sequence, the right to determine the negotiation sequence and whether to form an alliance. Therefore, we first construct decentralized and centralized decision-making models under the dual-credit policy, obtains the optimal strategy under different decision modes, and analyzes them. Then, considering suppliers can form supplier alliance to cooperate with the manufacturer, we construct bargaining game models under the conditions of the non-alliance and alliance of suppliers, and discuss the coordination strategy of supply chain. We analyzed the impact of negotiating power, negotiation sequence and the right to determine the negotiation sequence, whether to form an alliance on parts prices and profits.

### Model building

We consider the market demand function of NEVs as follows $q=a-p+\theta (T_{m}+\sum_{i=1}^{n}T_{i})$ where *a* is the potential demand of the market, *p* is the sales price of NEVs, and *θ* is the preference coefficient of consumers on NEVs technology level. The greater the coefficient *θ*, the greater impact of NEVs technology level on market demand. The subscript *i* denotes a supplier, *i* = 1, 2 ⋯, *n*, and the subscript *m* denotes a manufacturer.

Under the dual-credit policy, credits become a new commercial resource that can be traded, and NEVs manufacturer’s profit is affected by NEVs credits revenue. It is assumed that under the dual-credit policy, each NEVs can obtain NEVs credit *ε* at the current technology level *T*_0_. Further, considering that manufacturer and suppliers carry out R&D to improve the technical level of NEVs to ${(T}_{m}+\sum_{i=1}^{n}T_{i})$, the NEVs credit that can be increased is $\lambda \left(T_{m}+\sum_{i=1}^{n}T_{i}-T_{0}\right)$, *λ* is the credit coefficient of NEVs technology improvement, and *λ* > 0. Therefore, the NEVs credits obtained by each NEV is $\lambda \left(T_{m}+\sum_{i=1}^{n}T_{i}-T_{0}\right)$. For simplicity and without loss of generality, let T_0_ = 0, ε = 0. Set *p*_*e*_ as the transaction price of NEVs credit, affected by the supply and demand of credits in the credit market, the credit revenue obtained by the manufacturer for producing NEVs *q* is: $\lambda p_{e}(T_{m}+\sum_{i=1}^{n}T_{i})q$.

We assume that the parts supplier’s R&D input cost is $\frac{1}{2}k_{i}T_{i}^{2}$, *k*_*i*_ > 0(*i* = 1, 2, ⋯*n*), where *k*_*i*_ is the R&D investment coefficient of suppliers. The automobile manufacturer’s R&D input cost is $\frac{1}{2}k_{m}T_{m}^{2}$, *k*_*m*_ > 0, where *k*_*m*_ is the R&D investment coefficient of the manufacturer. Both the manufacturer and supplier’s cost of production is set to 0.

Therefore, the supplier’s profit consists of parts sales revenue and R&D input cost; The manufacturer’s profit consists of three parts: sales revenue of NEVs, credits revenue and R&D input cost.

Based on the above description and hypothesis, the profit function of suppliers can be obtained as follows

$$\pi_{i}=w_{i}q-\frac{1}{2}k_{i}T_{i}^{2}$$ (1)

The profit function of manufacturers is

$$\pi_{m}=\omega q+p_{e}\lambda (T_{m}+\sum_{i=1}^{n}T_{i})q-\frac{1}{2}k_{m}T_{m}^{2}$$ (2)

Where: $p=\omega +\sum_{i=1}^{n}w_{i}$, *w*_*i*_ is the parts price of the supplier, *ω* is the manufacturer’s profit margin.

## Model analysis

### Optimal solution of decentralized decision

When the NEVs supply chain makes decentralized decision after the implementation of dual-credit policy (indicated with the superscript D), the manufacturer and N suppliers all aim to maximize their own profits, decide R&D investment and prices through the Stackelberg game. The decision-making process of the supply chain is divided into two stages: in the first stage, the manufacturer decides its own R&D investment and the sales price of NEVs according to the price of parts, R&D investment, and dual-credit policy. In the second stage, N suppliers decide their R&D investment and parts price.

In this part, we use the reverse induction method to solve the optimal solution of the NEVs supply chain.

Firstly, the supplier decides the R&D investment and price of the parts with the aim of maximizing its own profit. Let *π*_*i*_ be differentiated by *w*_*i*_ and *T*_*i*_ respectively; simultaneously solving $\frac{\partial \pi_{i}}{\partial w_{i}}=0$ and $\frac{\partial \pi_{i}}{\partial T_{i}}=0$, we can get $w_{i}^{D*}=\frac{\text{Z}(a-\omega +\theta T_{m})}{(n+1)\text{Z}-\theta^{2}\text{M}}$, $T_{i}^{D*}=\frac{\text{Z}(a-\omega +\theta T_{m})}{(n+1)\text{Z}-\theta^{2}\text{M}}$.

Where: $\text{M}=\sum_{\delta =1}^{n}\frac{\prod_{i=1}^{n}k_{i}}{k_{\delta }}k_{m}$, $\text{Z}=\prod_{i=1}^{n}k_{i}$.

Then, manufacturers decide the R&D investment and the sales price of NEVs with the aim of maximizing their own profits. Substituting $w_{i}^{D*}$, $T_{i}^{D*}$ into Eq (2), let *π*_*m*_ be differentiated by *ω* and *T*_*m*_ respectively; simultaneously solving $\frac{\partial \pi_{m}}{\partial \omega }=0$ and $\frac{\partial \pi_{m}}{\partial T_{m}}=0$, we can get $\omega^{D*}=\frac{a\left(\left(n+1\right)k_{m}\text{Z}-\lambda \left(\theta +p_{e}\lambda \right)\text{Z}-\theta \left(\theta +2p_{e}\lambda \right)\text{M}\right)}{\text{Z}\left(2nk_{m}+\text{N}\right)-2\theta \left(\theta +p_{e}\lambda \right)\text{M}}$, $T_{m}^{D*}=\frac{a\text{Z}\left(\vartheta +p_{e}\lambda \right)}{\text{Z}\left(2nk_{m}+\text{N}\right)-2\theta \left(\theta +p_{e}\lambda \right)\text{M}}$, $p^{D*}=\omega^{D*}+\sum_{i=1}^{n}w_{i}^{D*}$.

Where: N = 2*k*_*m*_ −(*θ* + *p*_*e*_*λ*)^2^.

Furthermore, the optimal strategy of the NEVs supply chain under decentralized decisions can be obtained as follows

$$\begin{array}{cc} T_{i}^{D*}=\frac{a\theta \text{Z}k_{m}}{k_{i}\left(\text{Z}\left(2nk_{m}+\text{N}\right)-2\theta \left(\theta +p_{e}\lambda \right)\text{M}\right)}, & \pi_{m}^{D*}=\frac{a^{2}k_{m}\text{Z}}{2\left(\text{Z}\left(2nk_{m}+\text{N}\right)-2\theta \left(\theta +p_{e}\lambda \right)\text{M}\right)} \end{array}$$

$$\begin{array}{cc} \pi_{i}^{D*}=\frac{a^{2}k_{m}^{2}{\text{Z}}^{2}\left(2k_{i}-\beta^{2}\right)}{2k_{i}{\left(\text{Z}\left(2nk_{m}+\text{N}\right)-2\theta \left(\theta +p_{e}\lambda \right)\text{M}\right)}^{2}}, & \pi^{D*}=\frac{a^{2}k_{m}\text{Z}\left(\text{Z}\left(4nk_{m}+\text{N}\right)-\beta \left(3\beta +2p_{e}\lambda \right)\text{M}\right)}{2{\left(\text{Z}\left(2nk_{m}+\text{N}\right)-2\theta \left(\theta +p_{e}\lambda \right)\text{M}\right)}^{2}}. \end{array}$$

When there is no dual-credit policy (indicated with the superscript ND), that is *p*_*e*_*λ* = 0. The optimal strategy of NEVs supply chain under decentralized decision is $T_{i}^{ND*}=\frac{a\theta \text{Z}k_{m}}{k_{i}\left(\text{Z}\left(2\left(n+1\right)k_{m}-\theta^{2}\right)-2\theta^{2}\text{M}\right)}$, $T_{m}^{ND*}=\frac{a\text{Z}\theta }{\text{Z}\left(2\left(n+1\right)k_{m}-\theta^{2}\right)-2\theta^{2}\text{M}}$, $I_{i}^{ND*}=\frac{{\left(a\theta \text{Z}k_{m}\right)}^{2}}{2k_{i}{\left(\text{Z}(2(n+1)k_{m}-\theta^{2})-2\theta^{2}\text{M}\right)}^{2}}$, $I_{m}^{ND*}=\frac{k_{m}{\left(a\text{Z}\theta \right)}^{2}}{2{\left(\text{Z}(2(n+1)k_{m}-\theta^{2})-2\theta^{2}\text{M}\right)}^{2}}$, $\pi_{m}^{ND*}=\frac{a^{2}k_{m}\text{Z}}{2(\text{Z}(2(n+1)k_{m}-\theta^{2})-2\theta^{2}\text{M})}$, $\pi_{i}^{ND*}=\frac{a^{2}k_{m}^{2}{\text{Z}}^{2}(2k_{i}-\theta^{2})}{2k_{i}{\left(\text{Z}(2(n+1)k_{m}-\theta^{2})-2\theta^{2}\text{M}\right)}^{2}}$, $\pi^{ND*}=\frac{a^{2}k_{m}\text{Z}(\text{Z}(4nk_{m}+\text{N})-3\theta^{2}\text{M})}{2{\left(\text{Z}(2(n+1)k_{m}-\theta^{2})-2\theta^{2}\text{M}\right)}^{2}}$, $q^{ND*}=\frac{a\text{Z}k_{m}}{\text{Z}(2(n+1)k_{m}-\theta^{2})-2\theta^{2}\text{M}}$.

### Optimal solution of centralized decision

When the NEVs supply chain makes centralized decision after the implementation of dual-credit policy (indicated with the superscript C), the members of the NEVs supply chain jointly decide the R&D investment and sales price of NEVs with the aim of maximizing the profit of the supply chain. The profit function of the NEVs supply chain is

$$\pi^{C}=pq+p_{e}\lambda (\sum_{i=1}^{n}T_{i}+T_{m})q-\frac{1}{2}\sum_{i=1}^{n}k_{i}T_{i}^{2}-\frac{1}{2}k_{m}T_{m}^{2}$$ (3)

Let *π*^*C*^ be differentiated by *T*_*m*_, *T*_*i*_, and *p* respectively; simultaneously solving $\frac{\partial \pi^{C}}{\partial T_{m}}=0$, $\frac{\partial \pi^{C}}{\partial T_{i}}=0$, and $\frac{\partial \pi^{C}}{\partial p}=0$, we can get $T_{i}^{C*}=\frac{{ak}_{m}\text{Z}(\theta +p_{e}\lambda )}{k_{i}(\text{ZN}-{\left(\theta +p_{e}\lambda \right)}^{2}\text{M})}$, $T_{m}^{C*}=\frac{a\text{Z}(\theta +p_{e}\lambda )}{\text{ZN}-{\left(\theta +p_{e}\lambda \right)}^{2}\text{M}}$.

Furthermore, the optimal strategy of the NEVs supply chain under centralized decisions can be obtained as follows

$$\pi^{C*}=\frac{a^{2}k_{m}\text{Z}}{2(\text{ZN}-{\left(\theta +p_{e}\lambda \right)}^{2}\text{M})},q^{C*}=\frac{a\text{Z}k_{m}}{\text{ZN}-{\left(\theta +p_{e}\lambda \right)}^{2}\text{M}}.$$

When there is no dual-credit policy (indicated with the superscript NC), that is *p*_*e*_*λ* = 0. The optimal strategy of NEVs supply chain under centralized decision is

$$T_{i}^{NC*}=\frac{{ak}_{m}\text{Z}\theta }{k_{i}\left(\text{Z}\left(2k_{m}-\theta^{2}\right)-\theta^{2}\text{M}\right)},T_{m}^{NC*}=\frac{a\text{Z}\theta }{\text{Z}\left(2k_{m}-\theta^{2}\right)-\theta^{2}\text{M}},I_{i}^{NC*}=\frac{{{(ak}_{m}\text{Z}\theta )}^{2}}{2k_{i}{\left(\text{Z}(2k_{m}-\theta^{2})-\theta^{2}\text{M}\right)}^{2}},I_{m}^{NC*}=\frac{k_{m}{\left(a\text{Z}\theta \right)}^{2}}{2{\left(\text{Z}(2k_{m}-\theta^{2})-\theta^{2}\text{M}\right)}^{2}},q^{NC*}=\frac{a\text{Z}k_{m}}{\text{Z}(2k_{m}-\theta^{2})-\theta^{2}\text{M}},\pi^{NC*}=\frac{a^{2}k_{m}\text{Z}}{2(\text{Z}(2k_{m}-\theta^{2})-\theta^{2}\text{M})}.$$

### Optimal solution analysis

In the previous part, we have obtained the optimal strategy of NEVs supply chain under centralized decision and decentralized decision respectively. This section will reveal the impact of dual-credit policy on the optimal strategy of NEVs supply chain and the problem of decision maladjustment under decentralized decision by comparing and analyzing suppliers’ R&D investment and supply chain profit under different circumstances.

**Proposition 1.** Under the dual-credit policy, the profit of NEVs supply chain increases, the R&D investment increases, the technical level of NEV increases, and the output increases.

**Proof of Proposition 1.** Substituting $I_{i}^{D*}$, $I_{i}^{ND*}$, $I_{m}^{D*}$, $I_{m}^{ND*}$, $I_{i}^{C*}$, $I_{i}^{NC*}$, $I_{m}^{C*}$, $I_{m}^{NC*}$, *q*^D^*, *q*^ND^*, *q*^C^*, *q*^NC^*, $T_{i}^{D*}$, $T_{i}^{ND*}$, $T_{m}^{D*}$, $T_{m}^{ND*}$, $T_{i}^{C*}$, $T_{i}^{NC*}$, $T_{m}^{C*}$, $T_{m}^{NC*}$, *π*^C^*, *π*^NC^*, *π*^ND^*, *π*^D^* into $I_{i}^{D*}-I_{i}^{ND*}$, $I_{m}^{D*}-I_{m}^{ND*}$, $I_{i}^{C*}-I_{i}^{NC*}$, $I_{m}^{C*}-I_{m}^{NC*}$, *q*^D^* − *q*^ND^*, *q*^C^* − *q*^NC^*, $T_{i}^{D*}-T_{i}^{ND*}$, $T_{i}^{C*}-T_{i}^{NC*}$, $T_{m}^{D*}-T_{m}^{ND*}$, $T_{m}^{C*}-T_{m}^{NC*}$, *π*^C^* − *π*^NC^*, *π*^D^* − *π*^ND^*, respectively. We can get $I_{i}^{D*}-I_{i}^{ND*}=\frac{{\left(a\theta Ζk_{m}\right)}^{2}}{2k_{i}{\left(Ζ\left(2nk_{m}+Ν\right)-2\theta \left(\theta +p_{e}\lambda \right)Μ\right)}^{2}}-\frac{{\left(a\theta Ζk_{m}\right)}^{2}}{2k_{i}{\left(Ζ(2(n+1)k_{m}-\theta^{2})-2\theta^{2}Μ\right)}^{2}}>0$, $I_{m}^{D*}-I_{m}^{ND*}=\frac{k_{m}{\left(aΖ\left(\theta +p_{e}\lambda \right)\right)}^{2}}{2{\left(Ζ\left(2nk_{m}+Ν\right)-2\theta \left(\theta +p_{e}\lambda \right)Μ\right)}^{2}}-\frac{k_{m}{\left(aΖ\theta \right)}^{2}}{2{\left(Ζ(2(n+1)k_{m}-\theta^{2})-2\theta^{2}Μ\right)}^{2}}>0$, $I_{i}^{C*}-I_{i}^{NC*}=\frac{{\left({ak}_{m}Ζ(\theta +p_{e}\lambda )\right)}^{2}}{2{\left(ΖΝ-{\left(\theta +p_{e}\lambda \right)}^{2}Μ\right)}^{2}}-\frac{{{(ak}_{m}Ζ\theta )}^{2}}{2k_{i}{\left(Ζ(2k_{m}-\theta^{2})-\theta^{2}Μ\right)}^{2}}>0$, $I_{m}^{C*}-I_{m}^{NC*}=\frac{k_{m}{\left(aΖ(\theta +p_{e}\lambda )\right)}^{2}}{2{\left(ΖΝ-{\left(\theta +p_{e}\lambda \right)}^{2}Μ\right)}^{2}}-\frac{k_{m}{\left(aΖ\theta \right)}^{2}}{2{\left(Ζ(2k_{m}-\theta^{2})-\theta^{2}Μ\right)}^{2}}>0$, $q^{D*}-q^{ND*}=\frac{aΖk_{m}}{Ζ\left(2nk_{m}+Ν\right)-2\theta \left(\theta +p_{e}\lambda \right)Μ}-\frac{aΖk_{m}}{Ζ(2(n+1)k_{m}-\theta^{2})-2\theta^{2}Μ}>0$, $q^{C*}-q^{NC*}=\frac{aΖk_{m}}{ΖΝ-{\left(\theta +p_{e}\lambda \right)}^{2}Μ}-\frac{aΖk_{m}}{Ζ\left(2k_{m}-\theta^{2}\right)-\theta^{2}Μ}>0$, $T_{i}^{D*}-T_{i}^{ND*}=\frac{a\theta Ζk_{m}}{k_{i}\left(Ζ\left(2nk_{m}+Ν\right)-2\theta \left(\theta +p_{e}\lambda \right)Μ\right)}-\frac{a\theta Ζk_{m}}{k_{i}\left(Ζ\left(2\left(n+1\right)k_{m}-\theta^{2}\right)-2\theta^{2}Μ\right)}>0$, $T_{i}^{C*}-T_{i}^{NC*}=\frac{ak_{m}Ζ\left(\theta +p_{e}\lambda \right)}{k_{i}\left(ΖΝ-{\left(\theta +p_{e}\lambda \right)}^{2}Μ\right)}-\frac{ak_{m}Ζ\theta }{k_{i}\left(Ζ\left(2k_{m}-\theta^{2}\right)-\theta^{2}Μ\right)}>0$, $T_{m}^{D*}-T_{m}^{ND*}=\frac{aΖ\left(\theta +p_{e}\lambda \right)}{Ζ\left(2nk_{m}+Ν\right)-2\theta \left(\theta +p_{e}\lambda \right)Μ}-\frac{aΖ\theta }{Ζ\left(2\left(n+1\right)k_{m}-\theta^{2}\right)-2\theta^{2}Μ}>0$, $T_{m}^{C*}-T_{m}^{NC*}=\frac{aΖ\left(\theta +p_{e}\lambda \right)}{ΖΝ-{\left(\theta +p_{e}\lambda \right)}^{2}Μ}-\frac{aΖ\theta }{Ζ\left(2k_{m}-\theta^{2}\right)-\theta^{2}Μ}>0$, $\pi^{C*}-\pi^{NC*}=\frac{a^{2}k_{m}Ζ}{2(ΖΝ-{\left(\theta +p_{e}\lambda \right)}^{2}Μ)}-\frac{a^{2}k_{m}Ζ}{2(Ζ(2k_{m}-\theta^{2})-\theta^{2}Μ)}>0$, $\pi^{D*}-\pi^{ND*}=\frac{a^{2}k_{m}Ζ(Ζ(4nk_{m}+Ν)-\theta (3\theta +2p_{e}\lambda )Μ)}{2{\left(Ζ(2nk_{m}+Ν)-2\theta (\theta +p_{e}\lambda )Μ\right)}^{2}}-\frac{a^{2}k_{m}Ζ(Ζ(4nk_{m}+Ν)-3\theta^{2}Μ)}{2{\left(Ζ(2(n+1)k_{m}-\theta^{2})-2\theta^{2}Μ\right)}^{2}}$.

Q.E.D.

Compared with no dual-credit policy, after the government implements the dual-credit policy, no matter which decision NEVs supply chain adopts, the profit and R&D investment of the supply chain will increase. Dual-credit policy can always motivate NEVs supply chain to increase R&D investment, and NEVs’ technical level will be continuously improved. This will push the NEVs supply chain to shift from "subsidy-dependent" for short-term gains to "technology-dependent" for core competitiveness. At the same time, NEVs production increased, NEVs supply chain profits increased, dual-credit policy to promote NEVs large-scale, sustainable development.

**Proposition 2.** Under the dual-credit policy, the higher the credit coefficient of technology improvement, the higher the credit transaction price, the more profit of supply chain, the more investment in R&D, the higher the level of NEVs technology and the more production.

**Proof of Proposition 2**. Let $I_{i}^{D*}$, $I_{m}^{D*}$, $T_{i}^{D*}$, $T_{m}^{D*}$, q^D^*, π^D^*, $I_{i}^{C*}$, $I_{m}^{C*}$, $T_{i}^{C*}$,$T_{m}^{C*}$, π^C^* be differentiated by *λ*, respectively. We can get $\frac{\partial I_{i}^{D*}}{\partial \lambda }=\frac{a^{2}k_{m}^{2}\theta^{2}Z^{2}\left(2\theta p_{e}M+2Zp_{e}\left(\theta +p_{e}\lambda \right)\right)}{{k_{i}\left(\left(\left(2n+2\right)k_{m}-{\left(\theta +p_{e}\lambda \right)}^{2}\right)Z-2\theta \left(\theta +p_{e}\lambda \right)M\right)}^{3}}>0$, $\frac{\partial I_{m}^{D*}}{\partial \lambda }=\frac{a^{2}{k_{m}Z}^{3}p_{e}\left(\left(2n+2\right)k_{m}+{\left(\theta +p_{e}\lambda \right)}^{2}\right)\left(\theta +p_{e}\lambda \right)}{{\left(\left(2nk_{m}+N\right)Z-2\theta \left(\theta +p_{e}\lambda \right)M\right)}^{3}}$, $\frac{\partial T_{i}^{D*}}{\partial \lambda }=\frac{2ak_{m}Z\theta \left(2p_{e}\theta M+2p_{e}Z\left(\theta +p_{e}\lambda \right)\right)}{{k_{i}\left(\left(2nk_{m}+N\right)Z-2\theta \left(\theta +p_{e}\lambda \right)M\right)}^{2}}>0$, $\frac{\partial T_{m}^{D*}}{\partial \lambda }=\frac{ap_{e}Z^{2}\left(\left(2n+2\right)k_{m}+{\left(\theta +p_{e}\lambda \right)}^{2}\right)}{{\left(\left(2nk_{m}+N\right)Z-2\theta \left(\theta +p_{e}\lambda \right)M\right)}^{2}}>0$, $\frac{\partial q^{D*}}{\partial \lambda }=\frac{2ak_{m}Z\left(M\theta p_{e}+p_{e}Z\left(\theta +p_{e}\lambda \right)\right)}{{\left(\left(2nk_{m}+N\right)Z-2\theta \left(\theta +p_{e}\lambda \right)M\right)}^{2}}$, $\frac{\partial \pi^{D}}{\partial \lambda }=\frac{a^{2}k_{m}Zp_{e}(\theta M+Z(\theta +p_{e}\lambda ))(2(1+3n)k_{m}-{\left(\theta +p_{e}\lambda \right)}^{2})Z-2M\theta (2\theta +p_{e}\lambda ))}{{\left(\left(\left(2n+2\right)k_{m}-{\left(\theta +p_{e}\lambda \right)}^{2}\right)Z-2\theta \left(\theta +p_{e}\lambda \right)M\right)}^{3}}>0$, $\frac{\partial I_{i}^{C*}}{\partial \lambda }=\frac{p_{e}{\left(ak_{m}Z\right)}^{2}(\theta +p_{e}\lambda )(2k_{m}Z+(M+Z){\left(\theta +p_{e}\lambda \right)}^{2})}{{\left(NZ-M{\left(\theta +p_{e}\lambda \right)}^{2}\right)}^{3}}>0$, $\frac{\partial I_{m}^{C*}}{\partial \lambda }=\frac{p_{e}k_{m}{\left(aZ\right)}^{2}\left(\theta +p_{e}\lambda \right)\left(2k_{m}Z+\left(M+Z\right){\left(\theta +p_{e}\lambda \right)}^{2}\right)}{{\left(NZ-M{\left(\theta +p_{e}\lambda \right)}^{2}\right)}^{3}},$

$\frac{\partial T_{i}^{C*}}{\partial \lambda }=\frac{{aZp}_{e}k_{m}(2k_{m}Z+(M+Z){\left(\theta +p_{e}\lambda \right)}^{2})}{k_{i}{\left(NZ-M{\left(\theta +p_{e}\lambda \right)}^{2}\right)}^{2}}>0$, $\frac{\partial T_{m}^{C*}}{\partial \lambda }=\frac{aZp_{e}\left(2k_{m}Z+\left(M+Z\right){\left(\theta +p_{e}\lambda \right)}^{2}\right)}{{\left(NZ-M{\left(\theta +p_{e}\lambda \right)}^{2}\right)}^{2}}>0$,$\frac{\partial q^{C*}}{\partial \lambda }=\frac{2aZk_{m}p_{e}(\theta +p_{e}\lambda )(M+Z)}{{\left(NZ-M{\left(\theta +p_{e}\lambda \right)}^{2}\right)}^{2}}>0$, $\frac{\partial \pi^{C}}{\partial \lambda }=\frac{2a^{2}p_{e}k_{m}Z(M+Z)(\theta +p_{e}\lambda )}{{\left(NZ-M{\left(\theta +p_{e}\lambda \right)}^{2}\right)}^{3}}>0$.

By the same token, we can get $\frac{\partial I_{i}^{D*}}{\partial p_{e}}>0$, $\frac{\partial I_{m}^{D*}}{\partial p_{e}}>0$, $\frac{\partial T_{i}^{D*}}{\partial p_{e}}>0$, $\frac{\partial T_{m}^{D*}}{\partial p_{e}}>0$, $\frac{\partial q^{D*}}{\partial p_{e}}>0$, $\frac{\partial \pi^{D}}{\partial p_{e}}>0$ $\frac{\partial I_{i}^{C*}}{\partial p_{e}}>0$, $\frac{\partial I_{m}^{C*}}{\partial p_{e}}>0$, $\frac{\partial T_{i}^{C*}}{\partial p_{e}}>0$, $\frac{\partial q^{C*}}{\partial p_{e}}>0$, $\frac{\partial \pi^{C}}{\partial p_{e}}>0$.

Q.E.D.

The optimal strategy of NEVs supply chain is affected by the factor of dual-credit policy. The higher the credit transaction price, the higher the credit coefficient of technology improvement, the higher the profit of NEVs supply chain, the higher the R&D investment, and the higher the output and technology level of NEVs. Therefore, it is particularly important for the government to set or regulate the credit coefficient of technology improvement and the credit transaction price.

**Proposition 3**. Under centralized decisions, the profit of NEVs supply chain, the R&D investment of manufacturer and the R&D investment of suppliers are all higher than under decentralized decisions.

**Proof of Proposition 3**. Substituting $T_{m}^{D*}$, $T_{m}^{C*}$,$T_{i}^{D*}$, $T_{i}^{C*}$, *π*^D^*, *π*^C^*, into $T_{m}^{C*}-T_{m}^{D*}$,$T_{i}^{C*}-T_{i}^{D*}$, *π*^C^* − *π*^D^*, respectively. We can get $T_{m}^{C*}-T_{m}^{D*}=\frac{a\text{Z}(\theta +p_{e}\lambda )(2{nk}_{m}\text{Z}-({p_{e}\lambda }^{2}+\theta^{2})\text{M})}{\left(\text{ZN}-{\left(\theta +p_{e}\lambda \right)}^{2}\text{M})(\text{Z}(2nk_{m}+\text{N})-2\theta (\theta +p_{e}\lambda )\text{M}\right)}>0$, $T_{i}^{C*}-T_{i}^{D*}=\frac{{ak}_{m}\text{Z}(\theta ((2{nk}_{m}\text{Z}-\theta^{2}\text{M})+p_{e}\lambda (c(2nk_{m}+\text{N})-2\theta (\theta +\lambda )\text{M}))}{k_{i}(\text{ZN}-{\left(\theta +p_{e}\lambda \right)}^{2}\text{M})(\text{Z}(2nk_{m}+\text{N})-2\theta (\theta +p_{e}\lambda )\text{M}))}>0$, $\pi^{C*}-\pi^{D*}=\frac{a^{2}\text{Z}k_{m}(4nk_{m}\text{Z}(nZ-\theta^{2}\text{M})+\lambda^{2}\text{M}(\text{Z}(4nk_{m}+\text{N})-\theta (3\theta +2p_{e}\lambda )+\theta^{4}{\text{M}}^{2})}{2{\left(\text{Z}(2nk_{m}+\text{N})-2\theta (\theta +p_{e}\lambda )\text{M}\right)}^{2}(\text{ZN}-{\left(\theta +p_{e}\lambda \right)}^{2}\text{M})}>0$.

Q.E.D.

Compared with centralized decision-making, when the supply chain makes decentralized decisions, both automobile manufacturers and parts suppliers reduce their R&D investment. At the same time, this reduces the profit of the NEVs supply chain, leading to decision maladjustment and profit loss for the system in the NEVs supply chain. Therefore, manufacturers are motivated to implement a coordination strategy to encourage suppliers to increase R&D investment, jointly improve the technical level of NEVs, and at the same time improve the overall profits of the NEVs supply chain and manufacturers’ profits.

### Coordination strategy based on bargaining game model

Considering that the total profit of the supply chain and the R&D investment of NEVs are better with decentralized decisions under centralized decision, it is necessary to design a coordination strategy to enable independent supply chain companies to achieve an optimal centralized decision strategy. Therefore, we design a profit allocation strategy based on the bargaining game model to realize the coordination of the NEVs supply chain.

Compared with decentralized decision-making, the profit increment obtained by centralized decision-making in NEVs supply chain is: $\Delta \pi =\pi^{C*}-\pi^{D*}=\frac{a^{2}\text{Z}k_{m}(4nk_{m}\text{Z}(nZ-\theta^{2}\text{M})+({p_{e}\lambda )}^{2}\text{M}(\text{Z}(4nk_{m}+\text{N})-\theta (3\theta +2p_{e}\lambda )+\theta^{4}{\text{M}}^{2})}{2{\left(\text{Z}(2nk_{m}+\text{N})-2\theta (\theta +p_{e}\lambda )\text{M}\right)}^{2}(\text{ZN}-{\left(\theta +p_{e}\lambda \right)}^{2}\text{M})}$. Therefore, the coordination of the supply chain can be realized as long as the profit increment Δ*π* is reasonably distributed through the bargaining game.

First, the NEVs supply chain makes centralized decisions and jointly decides the R&D investment and sales price of NEVs. Then, the manufacturer and the suppliers decide the price of the parts and profit through the bargaining game.

### Supplier non-aligned negotiations

In the case of supplier non-aligned, the automobile manufacturer and N parts suppliers decide the distribution of profit increment through negotiation, represented by *π*_*m*_ and *π*_*i*_, respectively. Obviously, $\pi_{m}+\sum_{i=1}^{n}\pi_{i}=\Delta \pi$. Because the manufacturer negotiates sequentially with N suppliers, the manufacturer negotiates with only one supplier at a time. In fact, automobile manufacturers and suppliers conduct N rounds of pair-to-pair bargaining negotiations, and each bargaining game is

$${Max(\pi_{m}-d_{m})}^{\alpha_{i}}{\left(\pi_{i}-d_{i}\right)}^{\beta_{i}}$$ (4)

s.t. (*π*_*m*_, *π*_*i*_) ≥ (*d*_*m*_, *d*_*i*_)

$$\pi_{m}+\pi_{i}\leq \Pi_{j}$$

where: the subscript *j* indicates the *j*^*th*^ round of the bargaining negotiation. Π_*j*_ is the profit that can be distributed between the two parties in the *j*^*th*^ round of negotiation, Π_1_ = Δπ, Π_*j*_ = Π_*j*−1_ − *π*_(*i*,*j*−1)_. (*d*_*m*_, *d*_*i*_) are the agreement points at which the negotiations breaks down; that is, when the profit obtained by the manufacturer and the supplier is less than (*d*_*m*_, *d*_*i*_) the negotiations between the two parties fails to reach an agreement. (*d*_*m*_, *d*_*i*_) are the profit that the manufacturer and the supplier *i* can get when the negotiation breaks down; it is also the retained benefits of manufacturers and suppliers participating in centralized supply chain decision-making. To simplify the analysis, *d*_*m*_ = *d*_*i*_ = 0 is considered in this study. *α*_*i*_(0 < *α*_*i*_ < 1) represents the manufacturer’s negotiating power over supplier *i*. *β*_*i*_(0 < *β*_*i*_ < 1) represents the negotiating power of supplier *i* over the manufacturer, and meets: *α*_*i*_ + *βi* = 1.

Based on the above hypothesis and description, the equilibrium solution of supplier *i* in the *j*^*th*^ round of negotiations can be obtained as follows: (*π*_(*m*,*j*)_, *π*_(*i*,*j*)_) = (*α*_*i*_Π_*j*_, *β*_*i*_Π_*j*_).

In order to get higher profits from supply chain cooperation, manufacturers and suppliers often give commit to each other before negotiations, declaring that the profit they achieve in the cooperation cannot be lower than $\bar{\pi_{\iota }}(\iota =1,2,\cdots ,n,m)$, otherwise they will quit the cooperation. However, these commitments are partial, and they can revoke their commitments at a cost of *c*_*l*_, which may be a loss of credibility or reputation. When the actual profit obtained through bargaining negotiations is no less than $\bar{\pi_{\iota }}$, there is no revoking cost. When the actual profit obtained through bargaining negotiations is lower than $\bar{\pi_{\iota }}$, it will result in a revoking cost; that is $c_{\iota }=\sigma_{\iota }\left(\bar{\pi_{\iota }}-\pi_{\iota }\right)$, where *σ*_*l*_ is the unit revoking cost, which satisfies *σ*_*l*_ > 0. Muthoo [26] assumed a linear revoking cost, given by

$$c_{\iota }=\left\{\begin{array}{cc} 0 & \bar{\pi_{\iota }}\leq \pi_{\iota }\\ \sigma_{\iota }\left(\bar{\pi_{\iota }}-\pi_{\iota }\right) & \bar{\pi_{\iota }}>\pi_{\iota } \end{array}\right.\;\left(\sigma_{\iota }>0\right)$$ (5)

In the case of ultimatums issued by the negotiating parties, when the participants issue commitment, Muthoo [25] has demonstrated that the equilibrium solution for the *j*^*th*^ round negotiations is: $\left(\pi_{\left(m,j\right)},\pi_{\left(i,j\right)}\right)=\left(\frac{1+\sigma_{m}}{2+\sigma_{m}+\sigma_{i}}\Pi_{j},\frac{1+\sigma_{i}}{2+\sigma_{m}+\sigma_{i}}\Pi_{j}\right)\left(i=1,2,\cdots ,n\right)$.

It can be seen from the equilibrium solution that, the larger *σ*_*l*_(*l* = 1, 2, ⋯, *n*, *m*) is, the greater the participant`s revoking cost, and the commitment is more credible. In the negotiations, the stronger the negotiating power, the greater the profit expected to be obtained in the negotiations. Muthoo [25] has proved that the relationship between the negotiating power of the participants and the revoking cost can be expressed as follows

$$\alpha_{i}=\frac{1+\sigma_{m}}{2+\sigma_{m}+\sigma_{i}}(i=1,2\cdots n)$$ (6)

$$\beta_{i}=\frac{1+\sigma_{i}}{2+\sigma_{m}+\sigma_{i}}(i=1,2\cdots n)$$ (7)

Since the profit obtained by each party through bargaining negotiations is related to its negotiating power, the negotiating power of each party is substituted into the *j*^*th*^ round of negotiations; thus, we can get the equilibrium solution (*π*_(*m*,*j*)_, *π*_(*i*,*j*)_). In the first round of negotiations, the profits of the manufacturer and suppliers, respectively are as follows $\alpha_{i}\Pi_{1}+\pi_{m}^{D*}$, $\beta_{i}\Pi_{1}+\pi_{i}^{D*}$; In the *j*^*th*^ round of negotiations, the profits of the manufacturer and suppliers are respectively, as follows $\alpha_{i}\Pi_{j}+\pi_{m}^{D*}$, $\beta_{i}\Pi_{j}+\pi_{i}^{D*}$.

**Proposition 4. The profit of suppliers increases with the improvement of their negotiating power.** Bringing the negotiation sequence forward can make the supplier obtain more profit, but the negotiation sequence does not affect the manufacturer’s profit.

**Proof of Proposition 4.** Let *π*_*i*_ be differentiated by *β*_*i*_, we can get $\frac{\partial \pi_{i}}{\partial \beta_{i}}=\Pi_{j}>0$, the profit of suppliers increases with the improvement of their negotiating power. In the (*j* − *ε*)^*th*^(*i* = 1, 2, ⋯, *n*; *j* = 2, 3, ⋯, *n*; *ε* ∈ {1, 2, ⋯, *j* − 1}) round of negotiations, the profits of supplier *i* are $\pi_{\left(i,j-\varepsilon \right)}=\beta_{i}\Pi_{j-\varepsilon }+\pi_{i}^{D*}$. In the *j*^*th*^ round of negotiations, the profits of supplier *i* are $\pi_{\left(i,j\right)}=\beta_{i}\Pi_{j}+\pi_{i}^{D*}$. By comparing the profit of supplier *i* in the *j*^*th*^ round with that of in the (*j* − *ε*)^*th*^ round of negotiations, we can see that Δ*π*_*i*_ = *β*_*i*_(Π_*j*−ε_ − Π_*j*_) = *β*_*i*_*α*_1_*α*_2_ ⋯ *α*_*j*−*ε*−1_(1 − *α*_*j*−*ε*_*α*_*j*−*ε*+1_⋯*α*_*j*−1_) > 0, meaning that suppliers in the *j*^*th*^ round of negotiations can gain more profit than in the (*j* − *ε*)^*th*^ round. The supplier can get more profits by bringing the negotiation sequence forward. Similarly, a delay in the sequence of negotiations will also lead to the loss of suppliers’ profits. Therefore, a supplier’s profit is not only affected by its own negotiating power, but also by the negotiation sequence. However, for the manufacturer, because he needs to participate in all rounds of the negotiations, the profit after the *j*^*th*^ round of negotiations is $\alpha_{i}\Pi_{j}+\pi_{m}^{D*}$, and the profit is only related to his negotiating power; and has nothing to do with the negotiation sequence.

Q.E.D.

When suppliers are not-aligned, manufacturers and N suppliers conduct N rounds of pair-to-pair negotiations, and by bringing the negotiation sequence forward, suppliers can obtain more profits. The stronger the negotiating power of suppliers, the more profits they can obtain by bringing the negotiation sequence forward. Correspondingly, the delay of negotiation sequence will cause the loss of suppliers’ profits, which is bound to lead suppliers to fight for the negotiation sequence to obtain more profits.

**Proposition 5.** Since the manufacturer has the right to determine the negotiation sequence, the supplier has to transfer the profits *P*_(*i*,*j*)_ to the manufacturer in order to get the *j*^*th*^ round of negotiations, *and* meets *P*(_*i*,*j*_) = *β*_*i*_(Π_*j*_ − Π_*n*_).

**Proof of Proposition 5.** The strategy of the supplier *i* is denoted as *ST*(_*i*,*j*_) = (*P*_(*i*,1)_, *P*_(*i*,2)_, ⋯, *P*_(*i*,*j*)_),where *P*_(*i*,*j*)_ is the profit transfer that the supplier needs to give to the manufacturer in order to obtain the *j*^*th*^ round of negotiations. We already know from proposition 2 that the profit of supplier *i* in the *j*^*th*^ round of the negotiations is $\beta_{i}\Pi_{j}+\pi_{i}^{D*}$. After transferring profits to the manufacturer, the actual profit they can get are $\beta_{i}\Pi_{j}-P_{\left(i,j\right)}+\pi_{i}^{D*}$. When supplier *i* is in the last round of negotiations, the profit obtained by supplier *i* is $\beta_{i}\Pi_{n}+\pi_{i}^{D*}$, which is the lowest profit obtained by supplier negotiations. This time, *P*_(*i*,*n*)_ = 0. In fact, the upper limit of the supplier’s transfer profit is achieved by transferring all the excess profit obtained by bringing the negotiation sequence forward to the manufacturer, so the supplier’s transfer profit *P*_(*i*,*j*)_ meets *P*_(*i*,*j*)_ = *β*_*i*_(Π_*j*_ − Π_*n*_). At the same time, the manufacturer will make full use of the supplier’s competition for the negotiation sequence, forcing the supplier to transfer profits to it, to obtain as much profit as possible.

Q.E.D.

The supplier can make more profit by bringing the negotiation sequence forward, and the manufacturer has the right to determine the negotiation sequence of the suppliers, so the supplier has to transfer profit to the manufacturer in order to win an advanced negotiation sequence; the more the supplier’s negotiation order is advanced, the more profit is given, and the maximum transferred profit is all the excess profit gained by the supplier due to the advanced negotiation sequence.

**Proposition 6.** The final profit of suppliers is $\pi_{i}^{*}=\beta_{i}\Pi_{n}+\pi_{i}^{D*}$; The final profit of the manufacturer is $\pi_{m}^{*}=\Pi_{1}-\sum_{i=1}^{n}\left(\beta_{i}\Pi_{n}\right)+\pi_{m}^{D*}$.

**Proof of Proposition 6.** As can be seen from proposition 3, after the supplier transfers profits to the manufacturer, the supplier can only be able to obtain the last round of the negotiations irrespective of which negotiation they participate in, which is the lowest profit that the supplier can get:$\pi_{i}^{*}=\beta_{i}\Pi_{j}-P_{\left(i,j\right)}+\pi_{i}^{D*}=\beta_{i}\Pi_{n}+\pi_{i}^{D*}$. The manufacturer’s profit is the sum of the negotiated profit plus the transfer of all suppliers. As a result, the manufacturer earns an excess profit by having the right to determine the sequence of negotiations:$\pi_{m}^{*}=\Pi_{1}-\sum_{i=1}^{n}\left(\beta_{i}\Pi_{n}\right)+\pi_{m}^{D*}$.

Q.E.D.

The manufacturer gets a higher profit distribution because it has the right to determine the negotiation sequence, and the manufacturer can even get the entire excess profit that the supplier gets due to bringing the negotiation sequence forward. In this case, the supplier can only be able to obtain the last round of the negotiations irrespective of which negotiation they participate in, which is the lowest profit that the supplier can get by participating in the negotiation.

**Proposition 7.** The profit of the supplier and the price of parts decrease with the increase in the negotiating power of the manufacturer. The profit of the supplier and the price of parts increases with the improvement of the manufacturer’s negotiating power over other suppliers.

**Proof of Proposition 7.** Substituting Π_1_ = Δ*π* and Π_*j*_ = Π_j−1_ − *π*_(i,j−1)_ into the profit function of supplier *i*, we can get $\pi_{i}^{*}=\beta_{i}\prod_{l=1}^{n}\alpha_{l}\Delta \pi +\pi_{i}^{D*}\left(i,l=1,2,3\cdots n,l\neq i\right)$, $w_{i}^{*}=\frac{\beta_{i}\prod_{i=1}^{i-1}\alpha_{n-i}\Delta \pi +\pi_{i}^{D*}+\frac{1}{2}k_{i}T_{i}^{2}}{q^{C*}}$. Let $\pi_{i}^{*}$, $w_{i}^{*}$ be differentiated by *α*_*i*_ and *α*_*ℓ*_ respectively; thus, we can get $\frac{{\partial w}_{i}^{*}}{\partial \alpha_{i}}=-\frac{\prod_{i=1}^{i-1}\alpha_{n-i}\Delta \pi }{q^{C*}}<0$, $\frac{\partial \pi^{C}}{\partial T_{i}}=0$, $\frac{{\partial w}_{i}^{*}}{\partial \alpha_{l}}=\frac{\beta_{i}\prod_{l=1}^{n-1}\alpha_{l-1}\Delta \pi }{q^{C*}}>0$, $\frac{\partial \pi_{i}^{*}}{\partial \alpha_{i}}=-\prod_{l=1}^{n}\alpha_{l}\Delta \pi <0$, $\frac{\partial \pi_{i}^{*}}{\partial \alpha_{l}}=\beta_{i}\prod_{l=1}^{n}\alpha_{l-1}\Delta \pi >0$.

Q.E.D.

When supplier *i* is in the *j*^*th*^ round of the negotiations, the manufacturer will demand higher profits at the negotiations due to the increase in the manufacturer’s negotiating power over supplier *i*. Therefore, the profit of supplier *i* decreases and the price of parts decreases. However, as the manufacturer’s negotiating power over other suppliers increases, the profits and the parts price of this supplier *i* increase. This is because when other suppliers negotiate in the (*j* − 1)^*th*^ round of negotiations, they negotiate before the negotiations of supplier. With the strengthening of the negotiating power of the manufacturer over other suppliers, the manufacturer will still demand higher profits, and it makes the *j*^*th*^ round profits available for increased distribution, supplier *i* will naturally demand a share of the increased profits from the manufacturer. If other suppliers participate in the (*j* + 1)^*th*^ round of negotiations, as the negotiating power of the manufacturer over other suppliers increases, the amount of profit of supplier *i* transferred to the manufacturer decreases, and so the profit of supplier *i* increases and the price of parts increase.

Therefore, irrespective of the round of negotiations in which supplier *i* participates, the price and profit of parts from supplier *i* will decrease with the increase in the manufacturer’s negotiating power over it, while they will increase with the increase in the manufacturer’s negotiating power over other suppliers.

### Supplier alliance negotiations

In the proof of propositions 4 and 5 but also affected by the negotiation sequence. By bringing the negotiation sequence forward, suppliers can get more profit. However, the supplier will have to transfer profits to the manufacturer in order to compete for the negotiation sequence, resulting in suppliers only being able to get the profits of the last round of the negotiations, which is the lowest profit that the supplier can get from the negotiations. In order to increase their profits, suppliers can form alliances to participate in supply chain cooperation. In reality, associations and unions are essentially alliances that, to some extent, help their members gain more benefit.

When suppliers form an alliance to participate in supply chain negotiations, the supplier alliance first bargains with the manufacturer to determine the profit distribution between the manufacturer and the supplier alliance, and then the alliance profits are distributed proportionally among the suppliers according to their bargaining power.

This paper considers that the negotiating power of the manufacturer over the supplier alliance is *α*_*s*_(0 < *α*_*s*_ < 1), and the negotiating power of the supplier alliance over the manufacturer is *β*_*s*_(0 < *β*_*s*_ < 1), and meets *β*_*s*_ + *α*_*s*_ = 1.

When the suppliers form more than one alliance, the supplier alliance will still transfer the profits to the manufacturer in order to compete for the negotiation sequence, resulting in the loss of the suppliers’ profits [27]. Therefore, only a grand alliance of all suppliers can solve the problem of manufacturers having the right to determine the negotiation sequence making excessive profits.

Based on the above analysis and assumptions, we consider that suppliers form a grand alliance to participate in NEVs supply chain cooperation. First, the supplier alliance and the manufacturer decide the profit distribution between the manufacturer and the supply chain alliance through bargaining and negotiations; the profit of the manufacturer is $\pi_{m}^{**}=\alpha_{s}\Delta \pi$, and the profit of the supplier alliance is $\pi_{s}^{**}=\beta_{s}\Delta \pi$. Then, suppliers distribute the profits of the alliance in proportion to their negotiating power. The profit that supplier *i* can get through alliance negotiations is $\pi_{i}^{**}=\frac{\beta_{i}}{\sum_{i=1}^{n}\beta_{i}}\pi_{s}^{**}+\pi_{i}^{D*}$.

**Proposition 8.** When the negotiating power of the supplier alliance meets $\beta_{s}\geq Max\{\sum_{i=1}^{n}\beta_{i}\prod_{l=1}^{n}\alpha_{l}\}(i,l=1,2,\cdots n,i\neq l)$, suppliers will form an alliance to negotiate with the manufacturer; otherwise, they will participate in the NEVs supply chain independently.

**Proof of Proposition 8.** The motivation of suppliers to form an alliance is to increase their profits from participating in supply chain cooperation. Therefore, when the profit obtained by all suppliers after the alliance is no lower than that obtained by separate negotiations, suppliers will choose an alliance. From proposition 6, we can know that when suppliers are non-aligned, the profit of suppliers is $\pi_{i}^{*}=\beta_{i}{Χ}^{n}+\pi_{i}^{D*}$. When the supplier forms a grand alliance to negotiate, the supplier’s profit is $\pi_{i}^{**}=\frac{\beta_{i}}{\sum_{i=1}^{n}\beta_{i}}\pi_{s}^{**}+\pi_{i}^{D*}=\frac{\beta_{i}}{\sum_{i=1}^{n}\beta_{i}}\beta_{s}\Delta \pi +\pi_{i}^{D*}$, As long as $\pi_{i}^{**}-\pi_{i}^{*}\geq 0$ is satisfied, the suppliers will form a grand alliance to negotiate with the manufacturer, that is, $(\frac{\beta_{i}}{\sum_{i=1}^{n}\beta_{i}}\beta_{s}-\beta_{i}\prod_{l=1}^{n}\alpha_{l})\Delta \pi \geq 0$ needs to be satisfied. Therefore, when $\beta_{s}\geq Max\{\sum_{i=1}^{n}\beta_{i}\prod_{l=1}^{n}\alpha_{l}\}(i,l=1,2,\cdots n,i\neq l)$, $\pi_{i}^{**}-\pi_{i}^{*}\geq 0$ is established, which can guarantee that the profits of all suppliers in the alliance negotiations are not lower than those in the separate negotiations. Otherwise, the profit after alliance is lower than that of non-alliance, and suppliers will choose to participate in supply chain cooperation independently.

Q.E.D.

Only when the negotiating power of the supplier alliance meets $\beta_{s}\geq Max\{\sum_{i=1}^{n}\beta_{i}\prod_{l=1}^{n}\alpha_{l}\}$ can suppliers increase their profits through an alliance, which is the condition for suppliers to form an alliance. The reason is that the purpose of forming a supplier alliance is to reduce the manufacturer`s excess profit gained by having the right to determine the negotiation sequence, and at the same time increase their own profit. Suppliers will choose to form a grand alliance only when their profits by forming an alliance are guaranteed to be no less than they would have been if they had negotiated separately; otherwise, they would choose not to form an alliance.

**Proposition 9.** The stronger the negotiating power of the supplier alliance, the greater the profit of supplier *i*, and the higher the price of parts $w_{i}^{**}$.

**Proof of Proposition 9.** Through alliance negotiations, the profit and parts price of the supplier are, respectively,$\pi_{i}^{**}=\frac{\beta_{i}}{\sum_{i=1}^{n}\beta_{i}}\beta_{s}\Delta \pi +\pi_{i}^{D*}$ and $w_{i}^{**}=\frac{\frac{\beta_{i}}{\sum_{i=1}^{n}\beta_{i}}\beta_{s}\Delta \pi +\pi_{i}^{D*}+\frac{1}{2}k_{i}T_{i}^{2}}{q^{C*}}$. Under centralized decisions in the supply chain, *k*_*i*_, *T*_*i*_ and *q*^C^* are all constant values; let $\pi_{i}^{*}$, $w_{i}^{*}$ be differentiated by *β*_*s*_; thus, we can get $\frac{\partial \pi_{i}^{**}}{\partial \beta_{s}}=\frac{\beta_{i}}{\sum_{i=1}^{n}\beta_{i}}\Delta \pi >0$, $\frac{\partial w_{i}^{**}}{\partial \Delta }=\frac{\frac{\beta_{i}}{\sum_{i=1}^{n}\beta_{i}}\Delta \pi }{q^{C*}}>0$.

Q.E.D.

When the supplier negotiates with the manufacturer through an alliance, the stronger the negotiating power of the alliance, the higher the price of the parts of the supplier, and the greater the profit.

## Numerical analyses

To intuitively illustrate the effectiveness of the coordination strategy and the impact of different values of parameters on the price of parts and the profits of all parties, correlation analysis is carried out through the following numerical examples. Taking the NEVs supply chain with three suppliers as an example, the parameters are as follows *k*_1_ = 10, *k*_2_ = 40, *k*_3_ = 30, *k*_*m*_ = 30, *θ* = 2, *λ* = 0.5 and a = 2000.

When parameters are substituted, the optimal strategy of the supply chain with decentralized decisions can be obtained as $T_{1}^{D*}=64.430$, $T_{2}^{D*}=16.107$, $T_{3}^{D*}=21.477$, $T_{m}^{D*}=26.846$, $\pi_{1}^{D*}=83023$, $\pi_{2}^{D*}=98590$, $\pi_{3}^{D*}=96860$, $\pi_{m}^{D*}=322148$, $\pi^{D*}=600622$ and *q*^D^* = 322.

The optimal strategy of the supply chain with centralized decisions is *π*^C^* = 2.49 × 10^6^, $T_{1}^{C*}=623.377$, $T_{2}^{C*}=155.844$, $T_{3}^{C*}=207.792$, $T_{m}^{C*}=207.792$ and *q*^C^* = 2494.

Obviously, compared with decentralized decision-making, when centralized decision-making is adopted, the R&D investment of suppliers and manufacturers, the number of new energy vehicles, and the profit of supply chain are all improved. The NEVs supply chain profit increase is Δ*π* = 1.89 × 10^6^.

Next, NEVs supply chain member enterprises distribute incremental profit Δ*π* through bargaining negotiations, and the unit revoking cost of the manufacturer and supplier *i* are, respectively, *σ*_*m*_ = 2, *σ*_1_ = 3, *σ*_2_ = 4 and *σ*_3_ = 3. By substituting into Eqs (6) and (7), the negotiating power of the manufacturer and supplier can be obtained *α*_1_ = 0.429, *α*_2_ = 0.375, *α*_3_ = 0.429, *β*_1_ = 0.571, *β*_2_ = 0.625 and *β*_3_ = 0.571.

When the supplier is non-aligned, the profits of supplier and manufacturer are, respectively, $\pi_{1}^{*}=256595$, $\pi_{2}^{*}=315554$, $\pi_{3}^{*}=270431$ and $\pi_{m}^{*}=164804$. When the supply chain makes centralized decisions and profits are distributed based on bargaining games, the profits of all parties in the supply chain are improved compared with the decentralized decision.

When the negotiating power of the supplier alliance is *β*_*s*_ = 0.370 > *max*{0.284, 0.325, 0.284} = 0.325, the conditions of the supplier alliance are met, and suppliers choose to form an alliance to participate in negotiations. Through the alliance negotiations, the profits obtained by suppliers are: $\pi_{1}^{**}=309060$, $\pi_{2}^{**}=345817$, $\pi_{3}^{**}=322896$ and $\pi_{m}^{**}=1512848$. Compared with the supplier before the alliance, through the alliance negotiations, the supplier’s profit is improved.

Therefore, the profit distribution strategy based on the bargaining game model on the one hand realizes R&D investment and the total profit of the supply chain under centralized decisions; on the other hand, it improves the profits of all members of the supply chain and realizes a "win-win" situation for the NEVs supply chain. Additionally, suppliers further improve their own profits by forming an alliance.

Next, we discuss the impact of the dual-credit policy factor, the negotiating power of suppliers and the number of suppliers on the NEVs supply chain.

### The impact of dual-credit policy factors

#### The impact of technology improvement credit coefficient on NEVs supply chain

**Fig 1:**
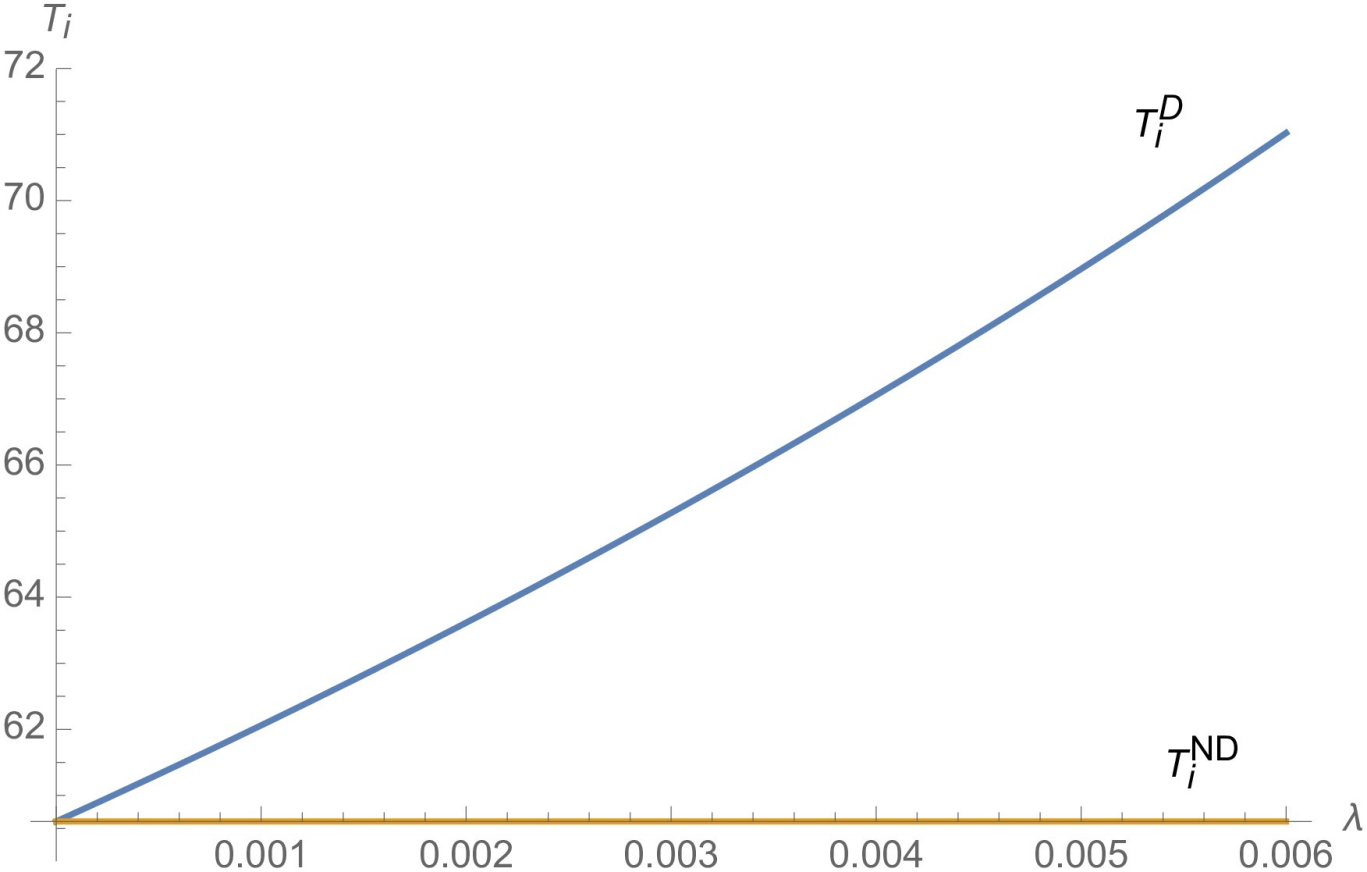
The impact of *λ* on $T_{i}^{D}$.

**Fig 2:**
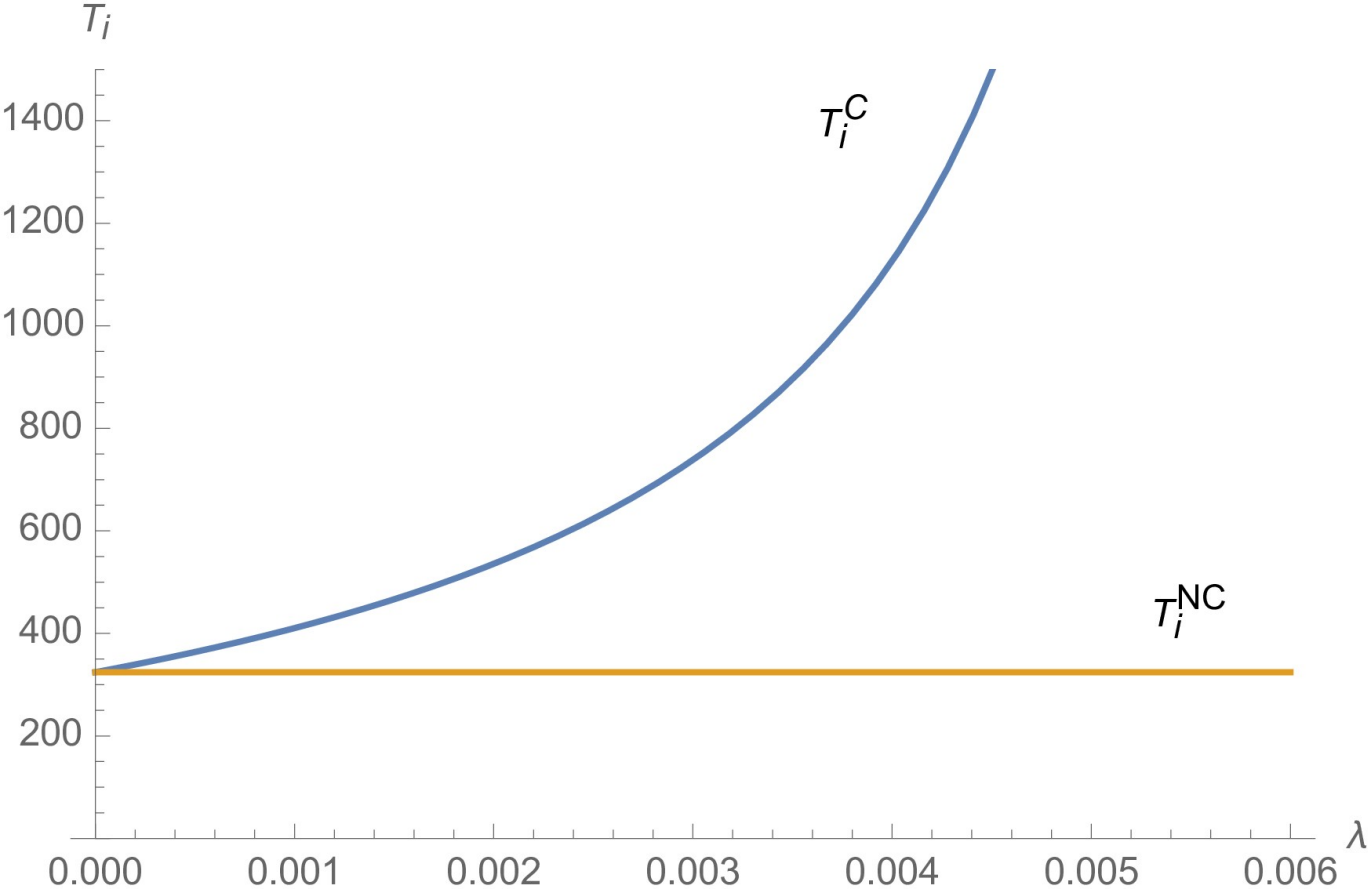
The impact of *λ* on $T_{i}^{C}$.

**Fig 3:**
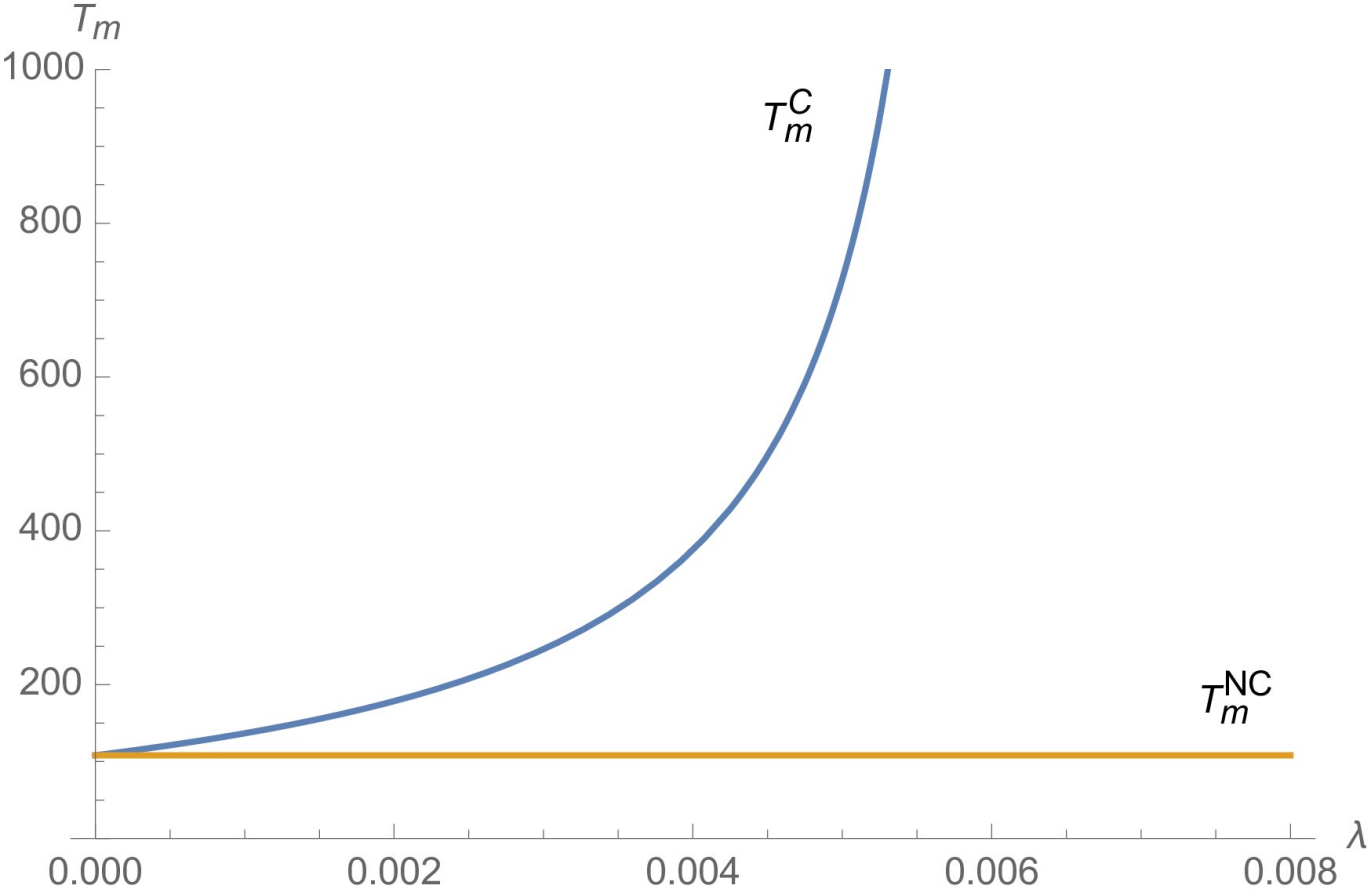
The impact of *λ* on $T_{m}^{C}$.

**Fig 4:**
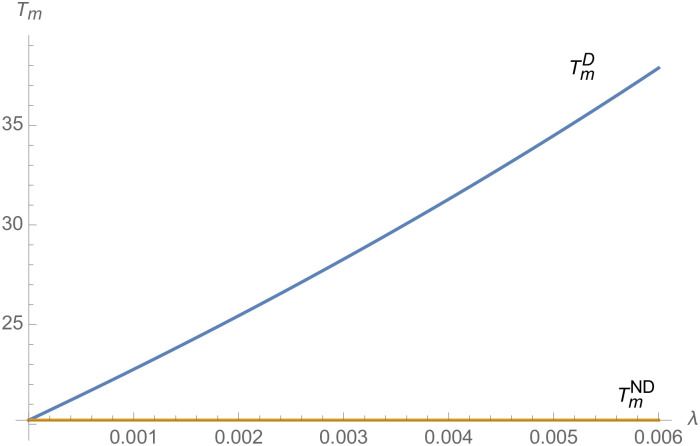
The impact of *λ* on $T_{m}^{D}$.

**Fig 5:**
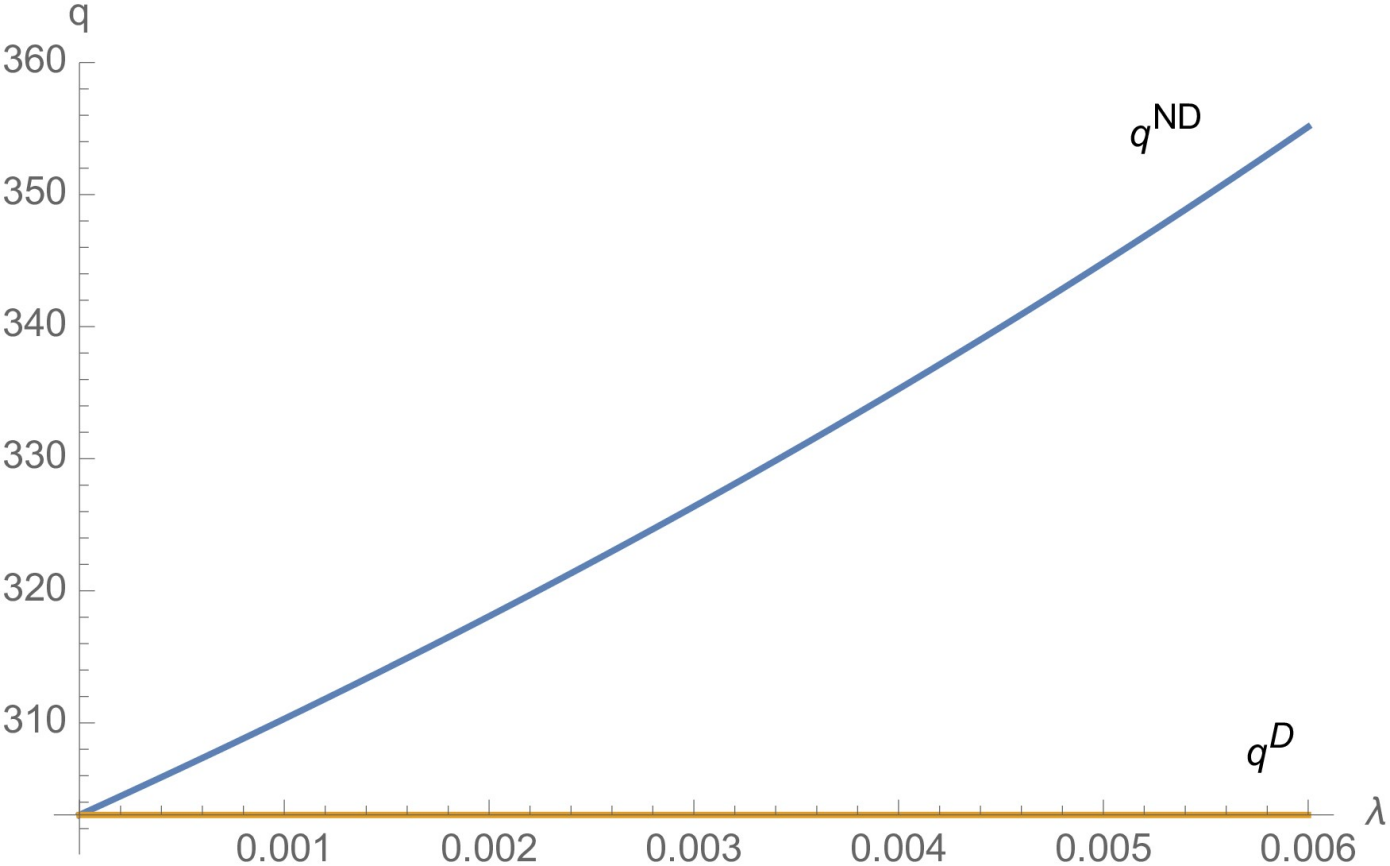
The impact of *λ* on *q*^*D*^.

**Fig 6:**
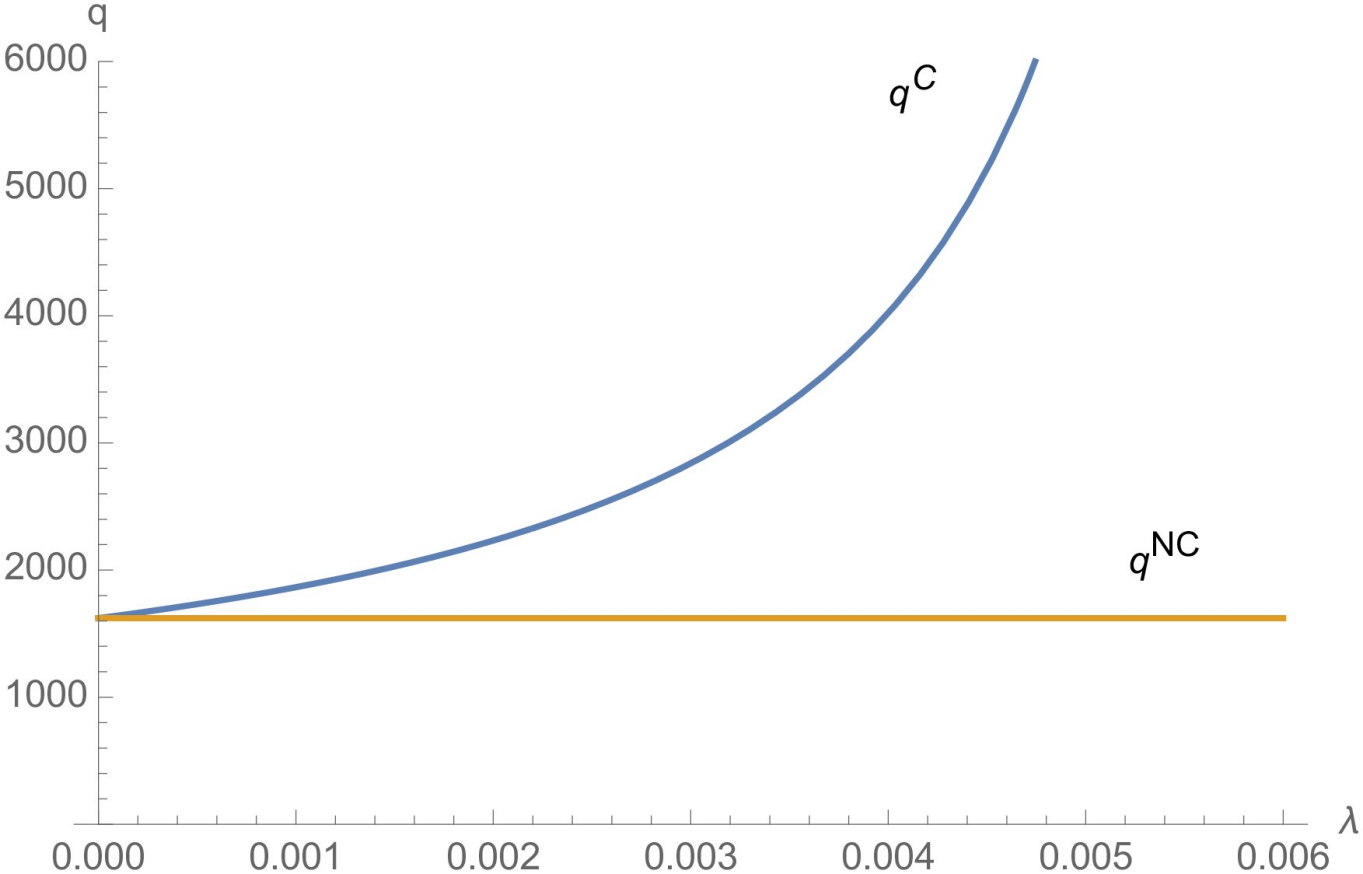
The impact of *λ* on *q*^*C*^.

**Fig 7:**
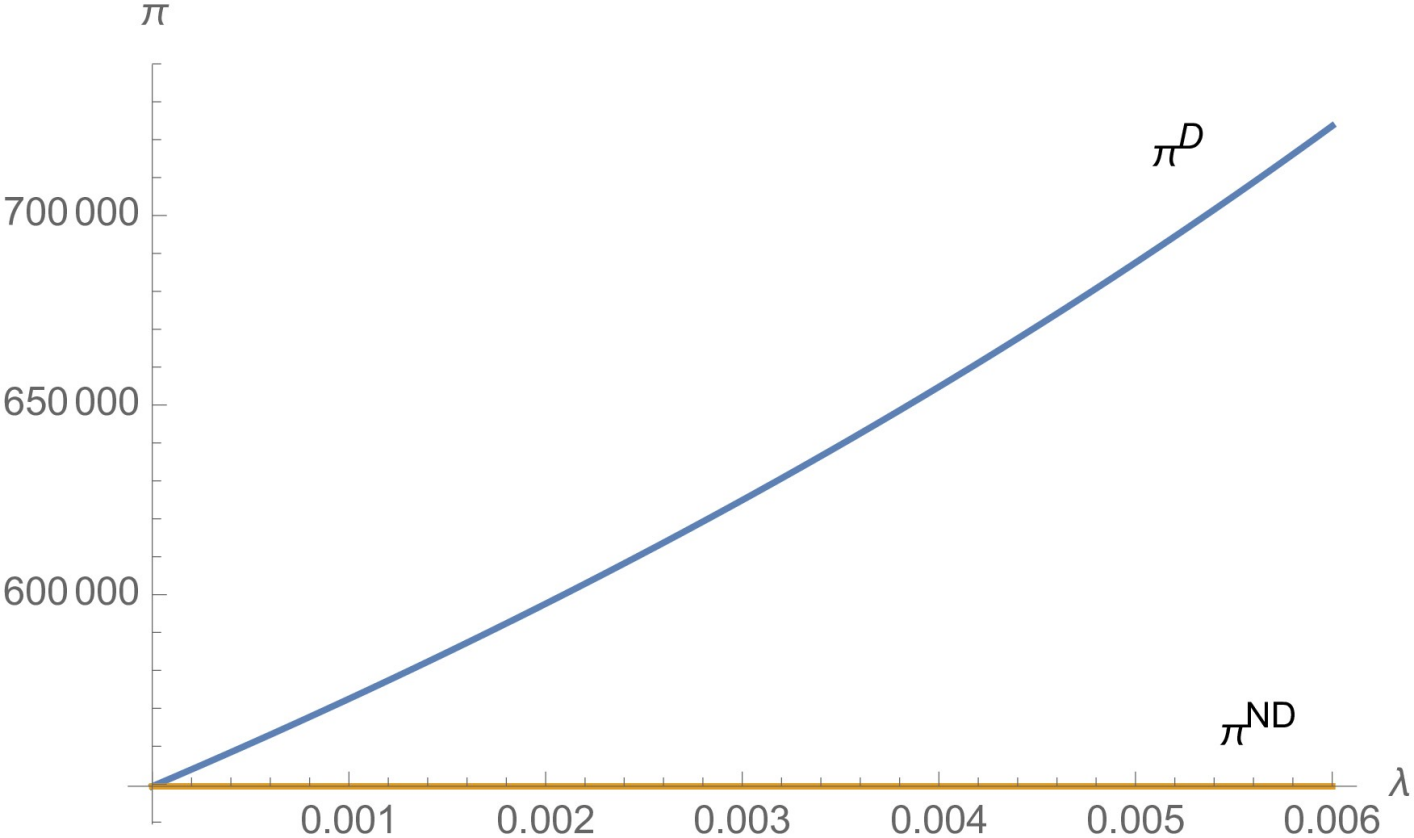
The impact of *λ* on *π*^*D*^.

**Fig 8:**
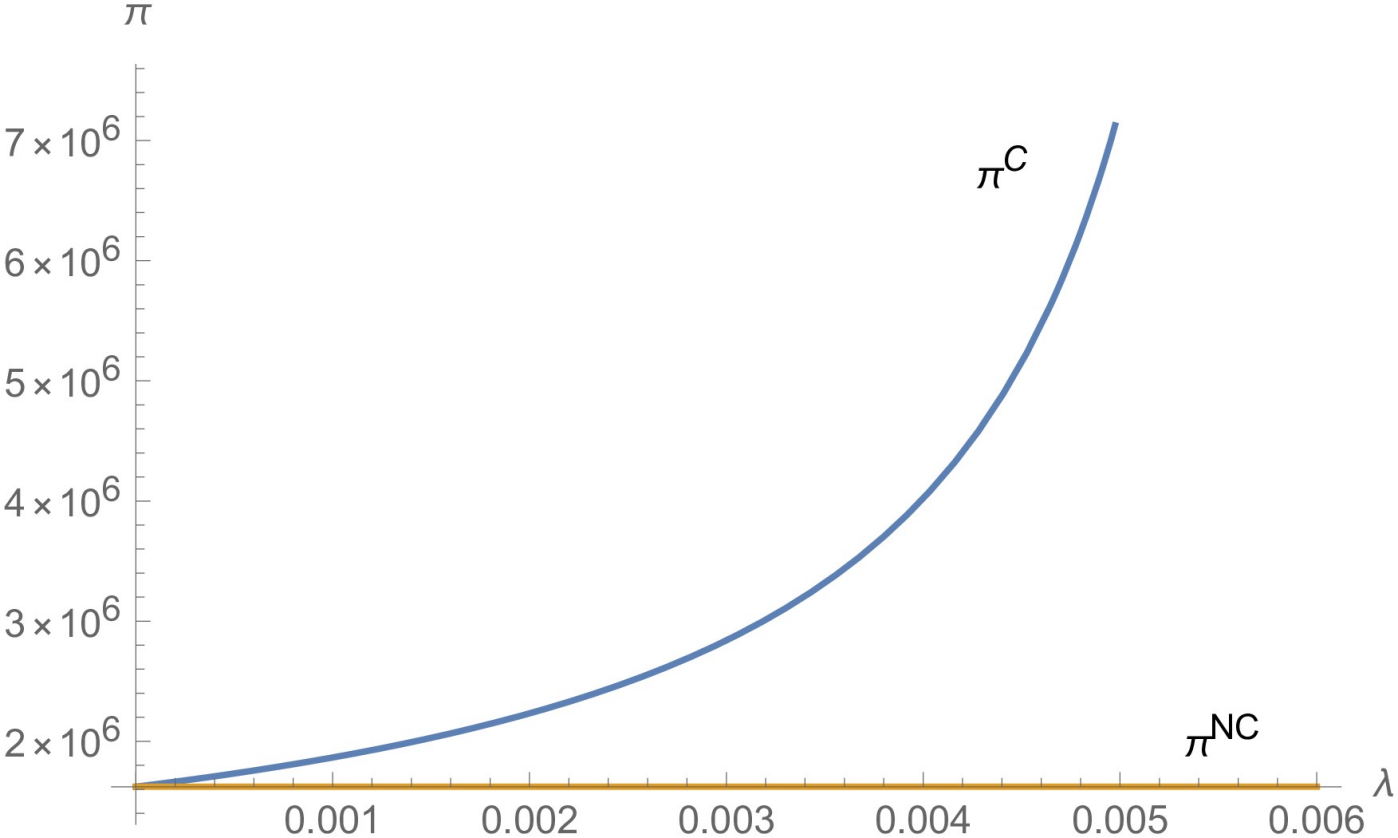
The impact of *λ* on *π*^*C*^.

**Fig 9:**
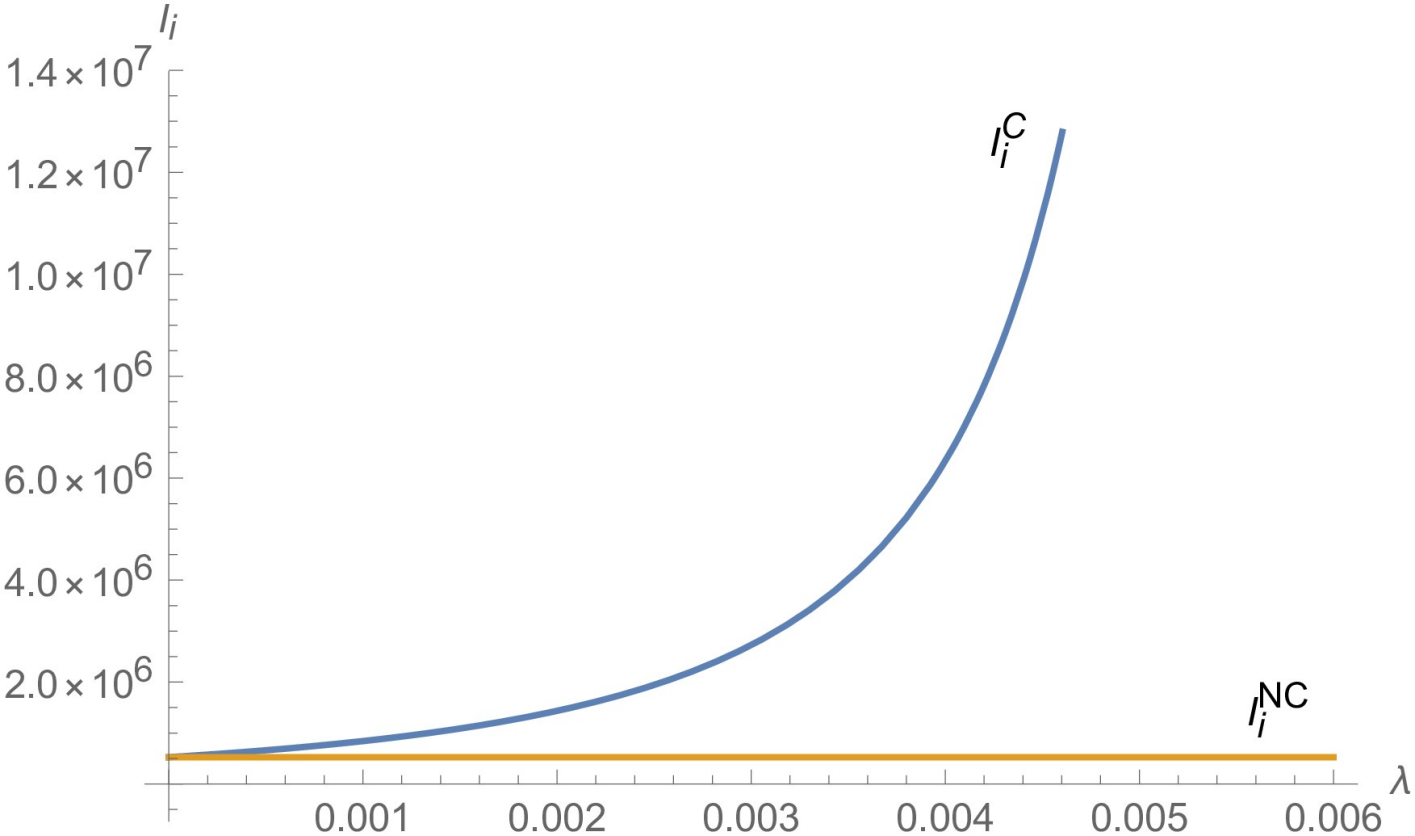
The impact of *λ* on $I_{i}^{C}$.

**Fig 10:**
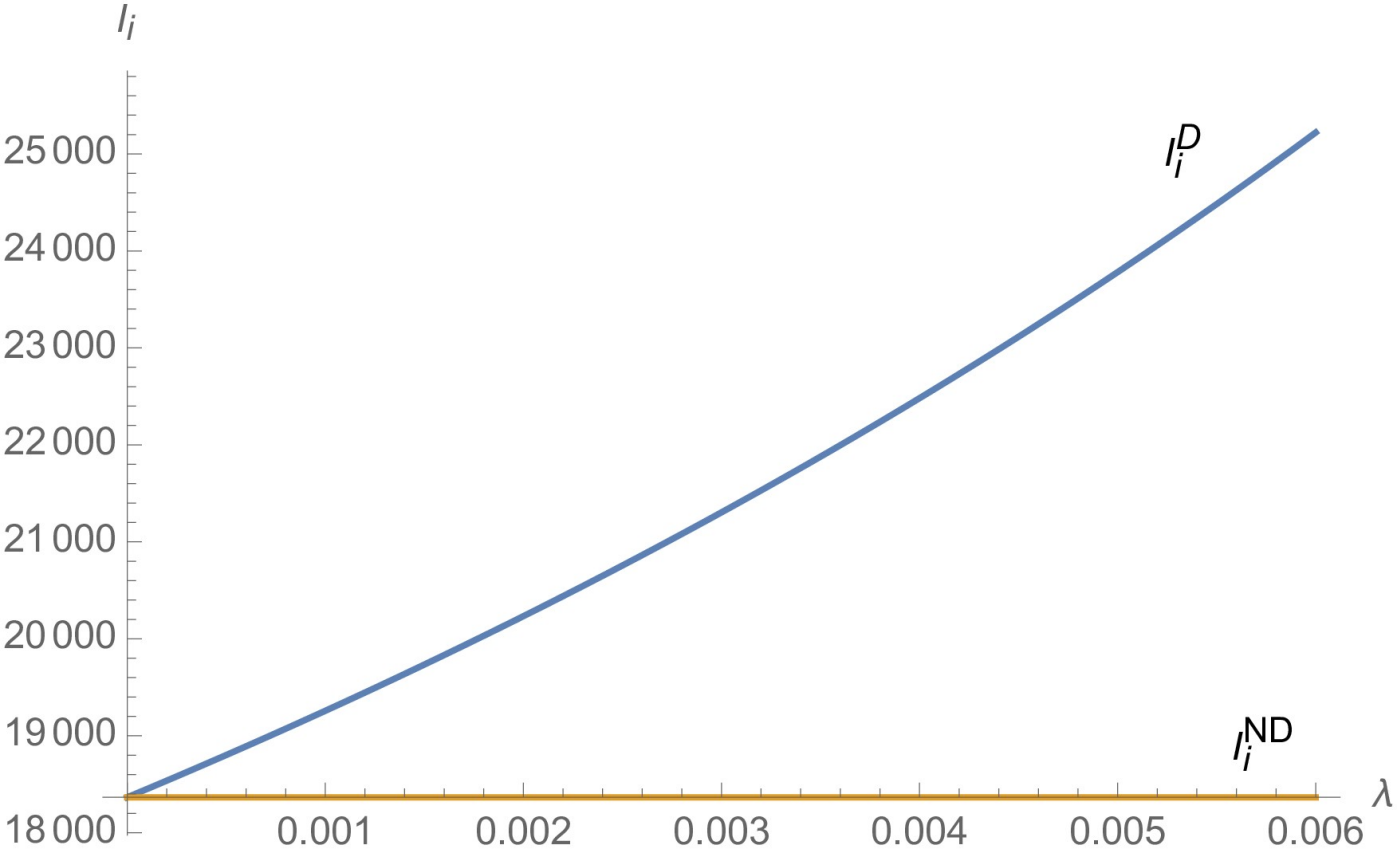
The impact of *λ* on $I_{i}^{D}$.

**Fig 11:**
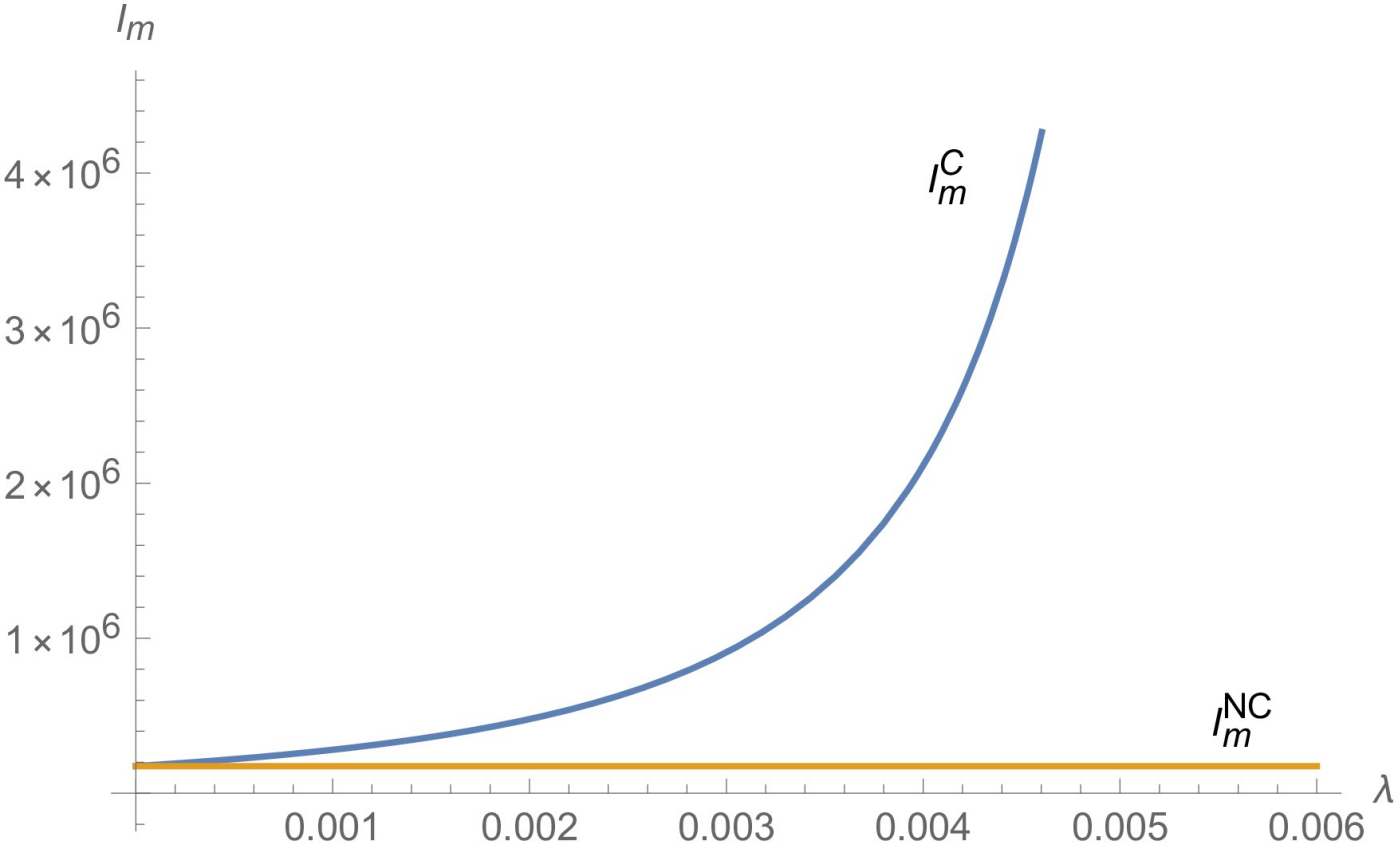
The impact of *λ* on $I_{m}^{C}$.

**Fig 12:**
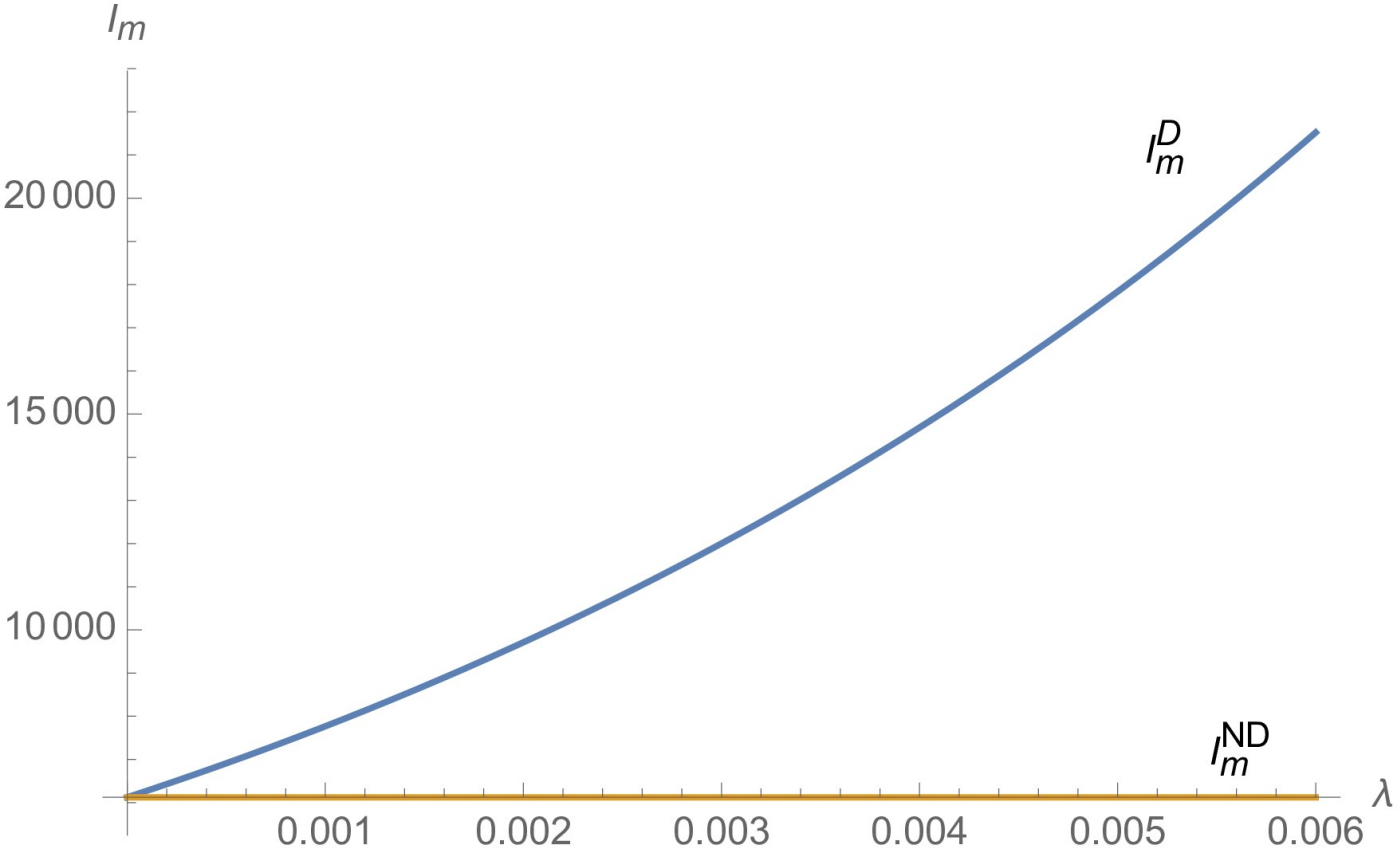
The impact of *λ* on $I_{m}^{D}$.

From Figs 1–4, compared with no dual-credit policy, the implementation of dual-credit policy improves the technical level of NEVs, no matter which decision-making mode is adopted in the NEVs supply chain. From Figs 5 and 6, the output of NEVs under both decentralized and centralized decisions increases after the implementation of dual-credit policy. From Figs 7 and 8, the profit of NEVs supply chain increases, after the implementation of dual-credit policy. From Figs 9–12, the R&D investment of manufacturer and suppliers increases, after the implementation of dual-credit policy.

At the same time, no matter which decision-making mode is adopted in the supply chain, with the improvement of the technology improvement credit, the profit of NEVs supply chain will increase, the R&D investment of manufacturer and suppliers will increase, and the output and technical level of NEVs will increase.

Therefore, the dual-credit policy promotes the improvement of NEV technology level, realizes the scale growth of NEVs, stimulates manufacturer and suppliers to increase R&D investment, and improves the profit of NEVs supply chain.

#### The impact of credit transaction price on NEVs supply chain

**Fig 13:**
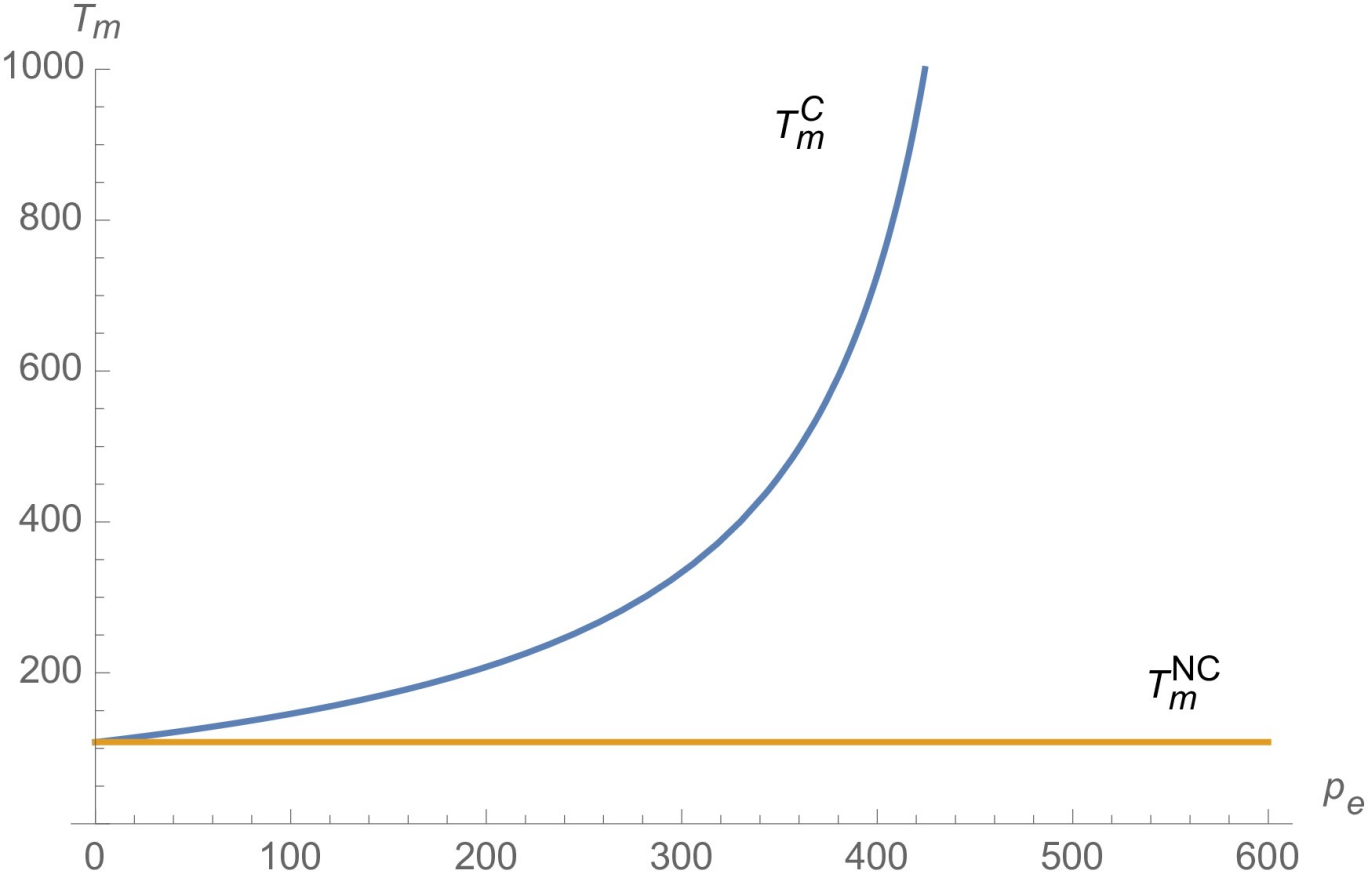
The impact of *p*_*e*_ on $T_{m}^{C}$.

**Fig 14:**
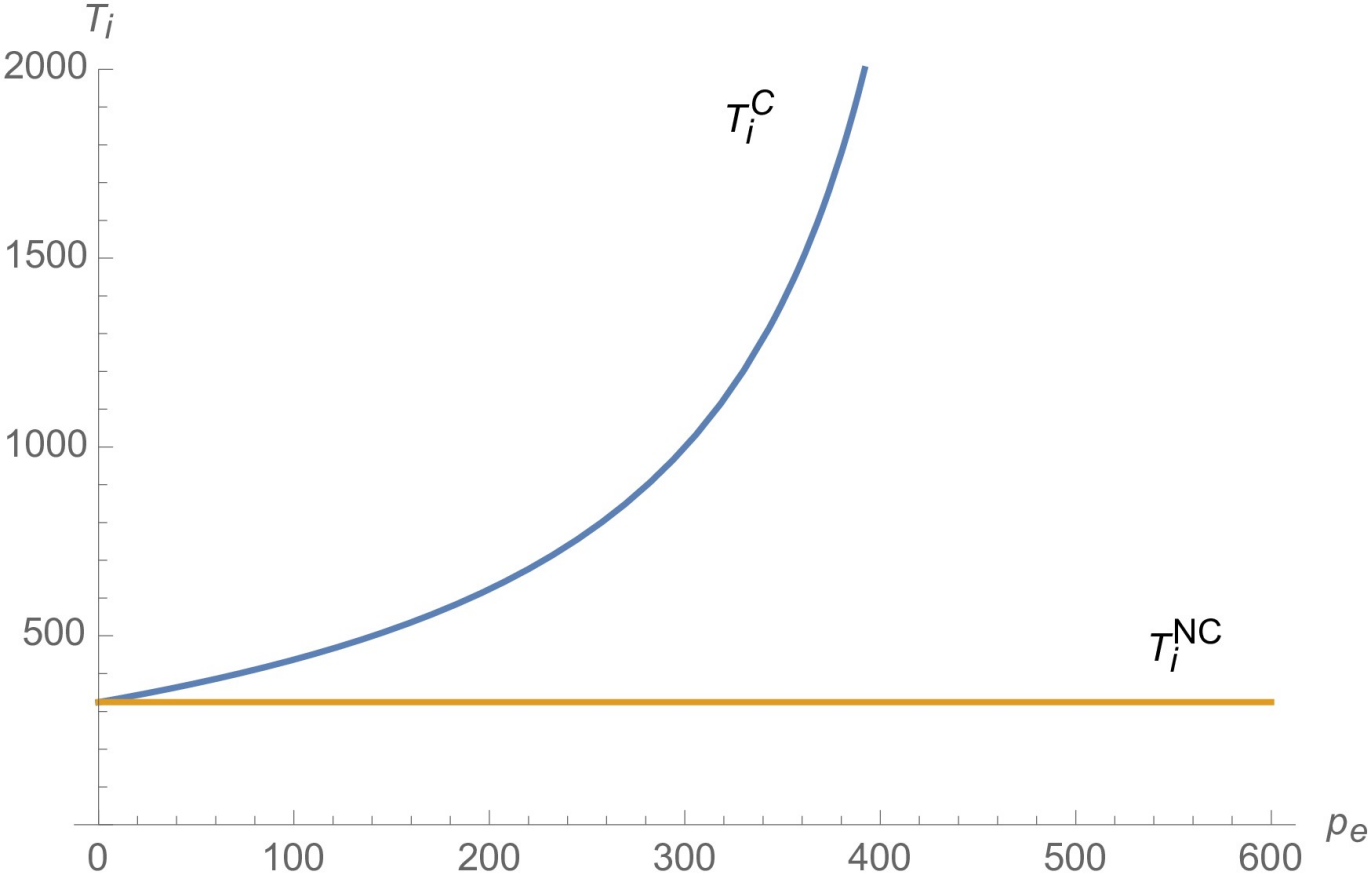
The impact of *p*_*e*_ on $T_{i}^{C}$.

**Fig 15:**
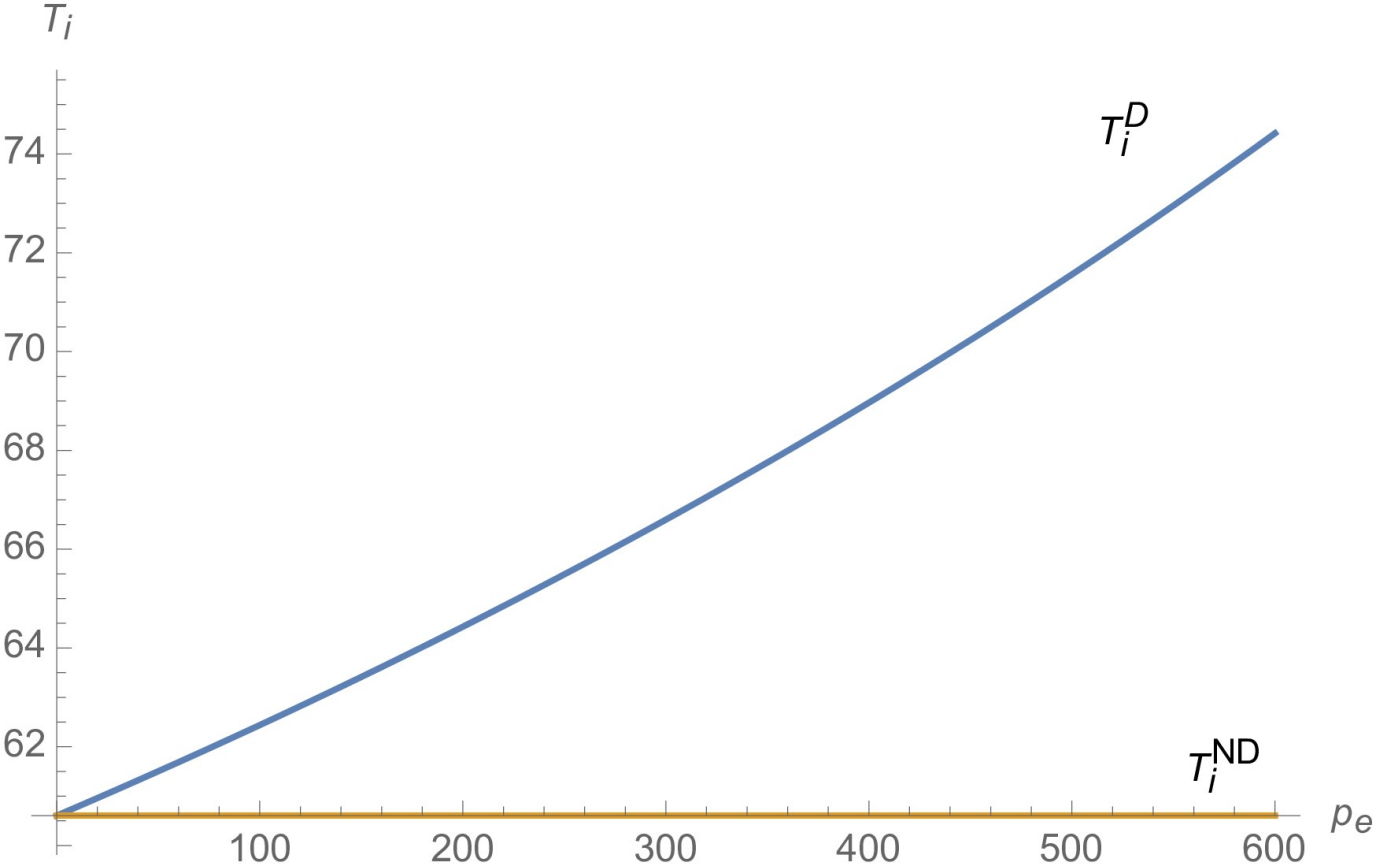
The impact of *p*_*e*_ on $T_{i}^{D}$.

**Fig 16:**
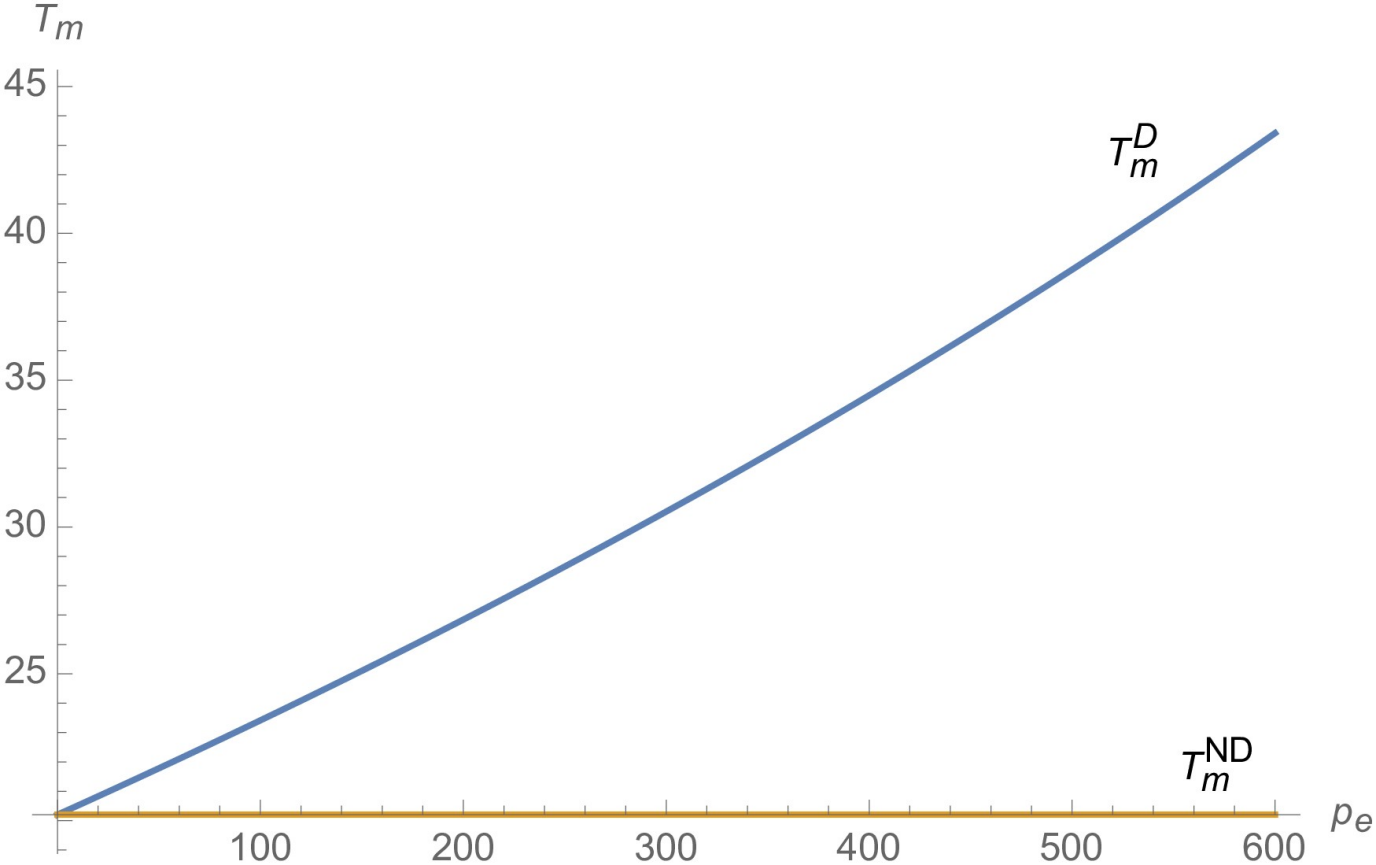
The impact of *p*_*e*_ on $T_{m}^{D}$.

**Fig 17:**
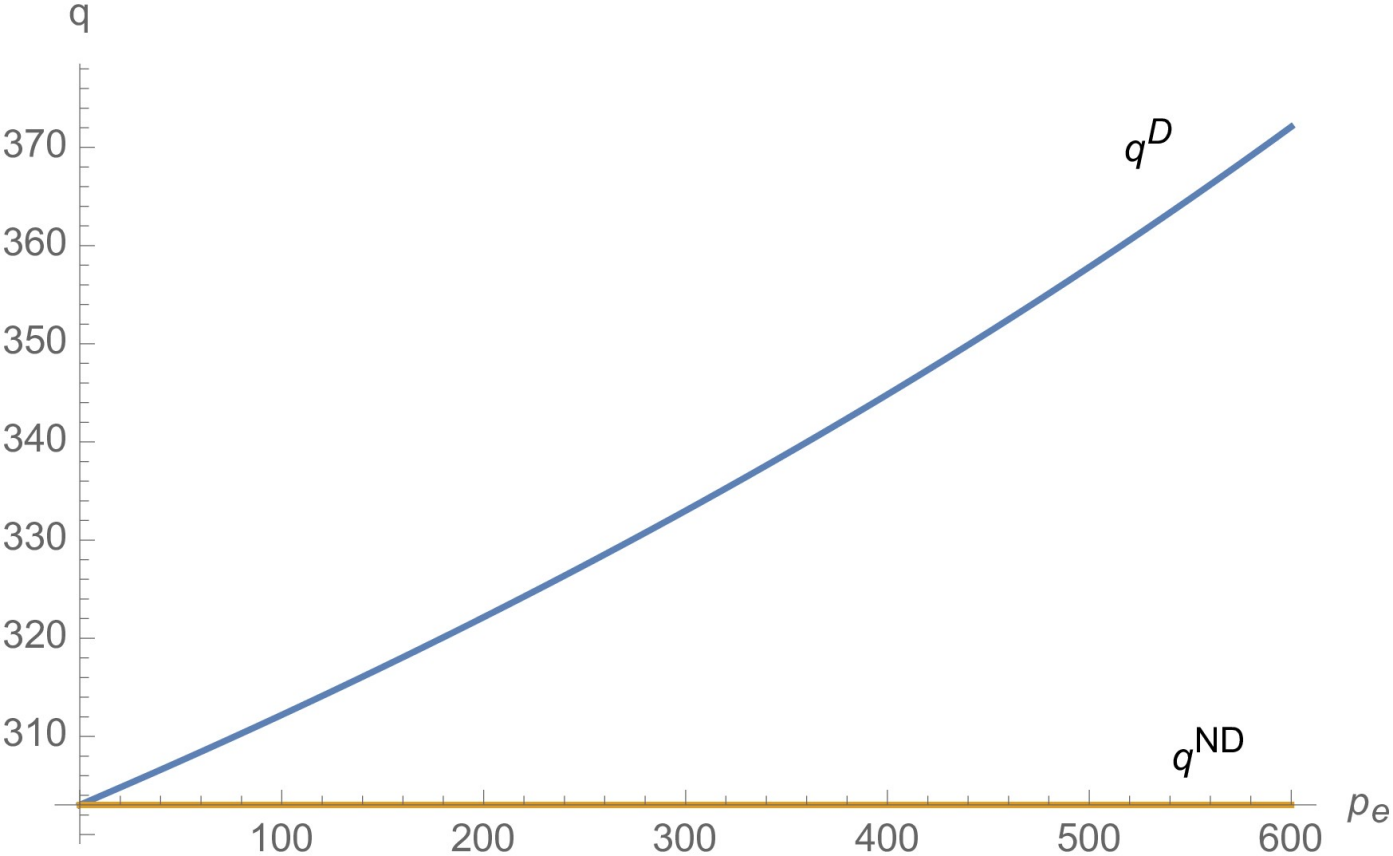
The impact of *p*_*e*_ on *q*^*D*^.

**Fig 18:**
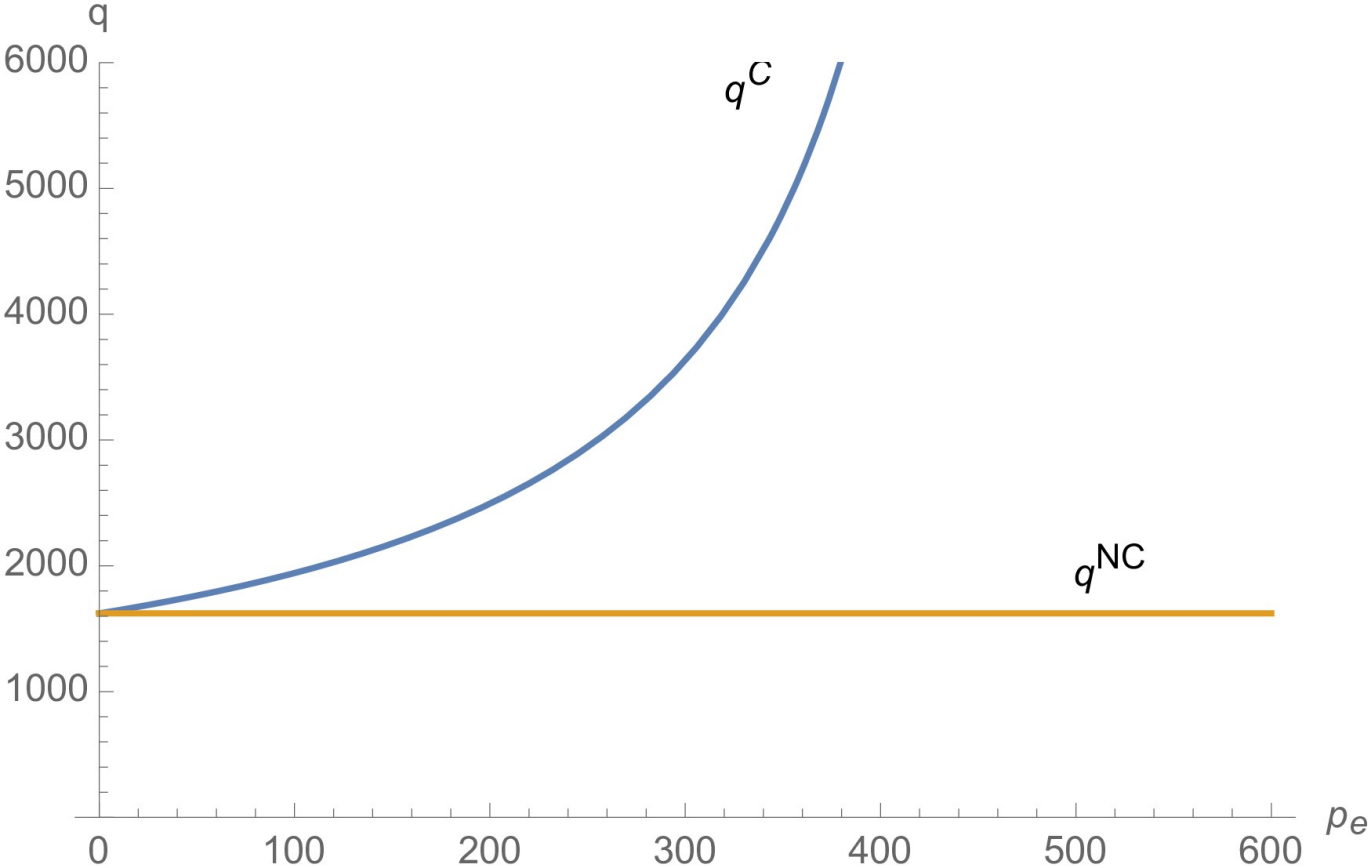
The impact of *p*_*e*_ on *q*^*C*^.

**Fig 19:**
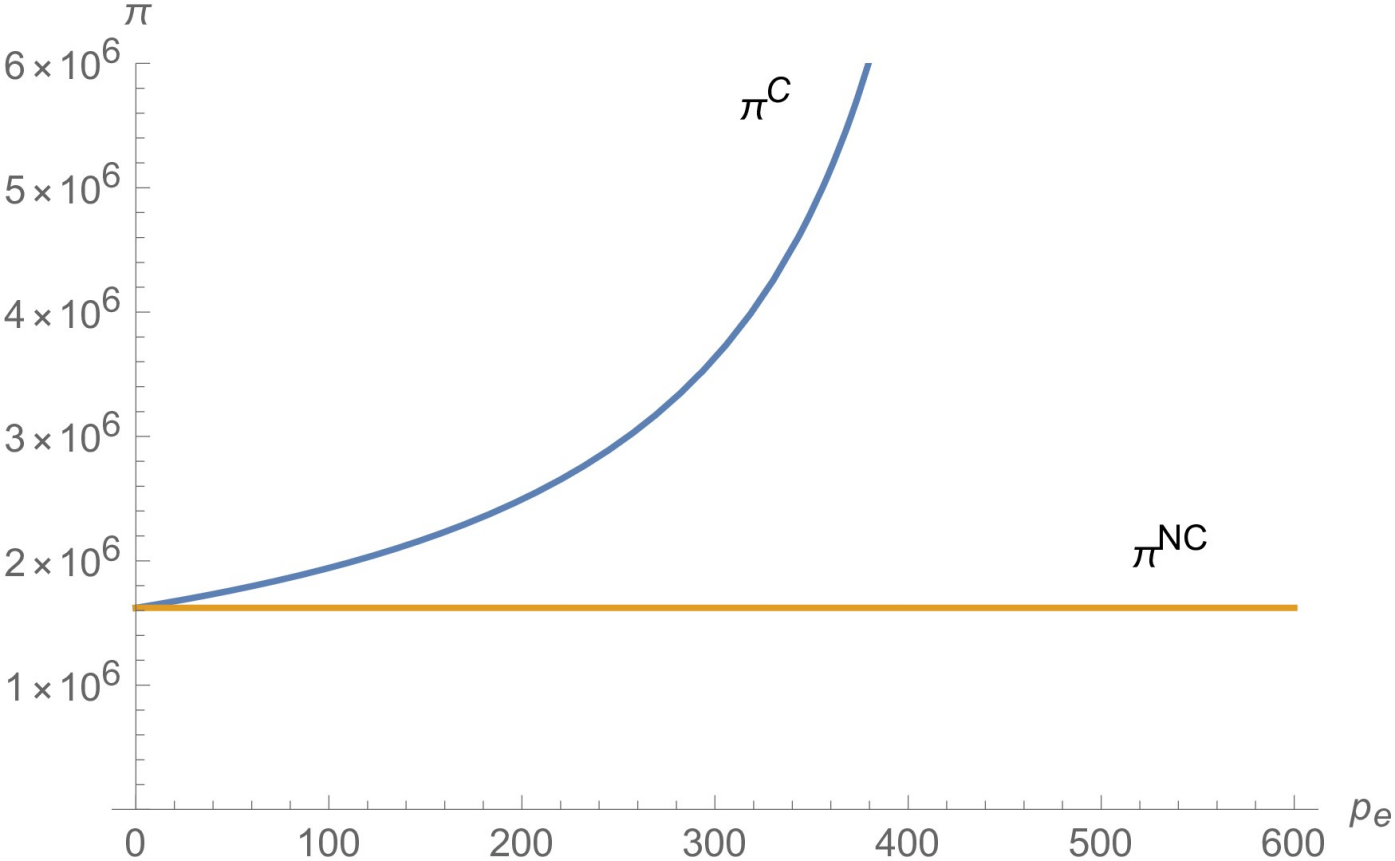
The impact of *p*_*e*_ on *π*^*C*^.

**Fig 20:**
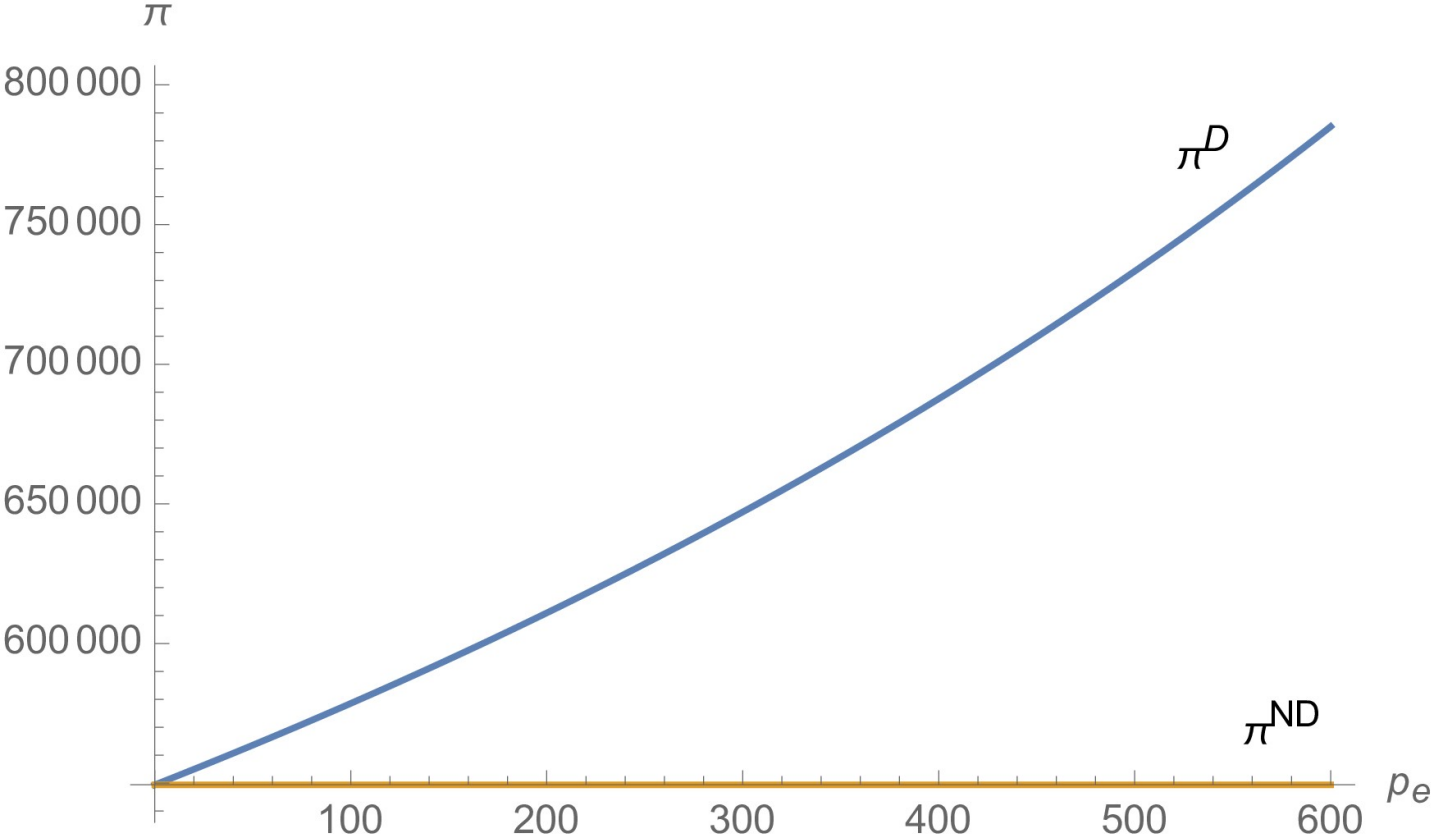
The impact of *p*_*e*_ on *π*^*D*^.

**Fig 21:**
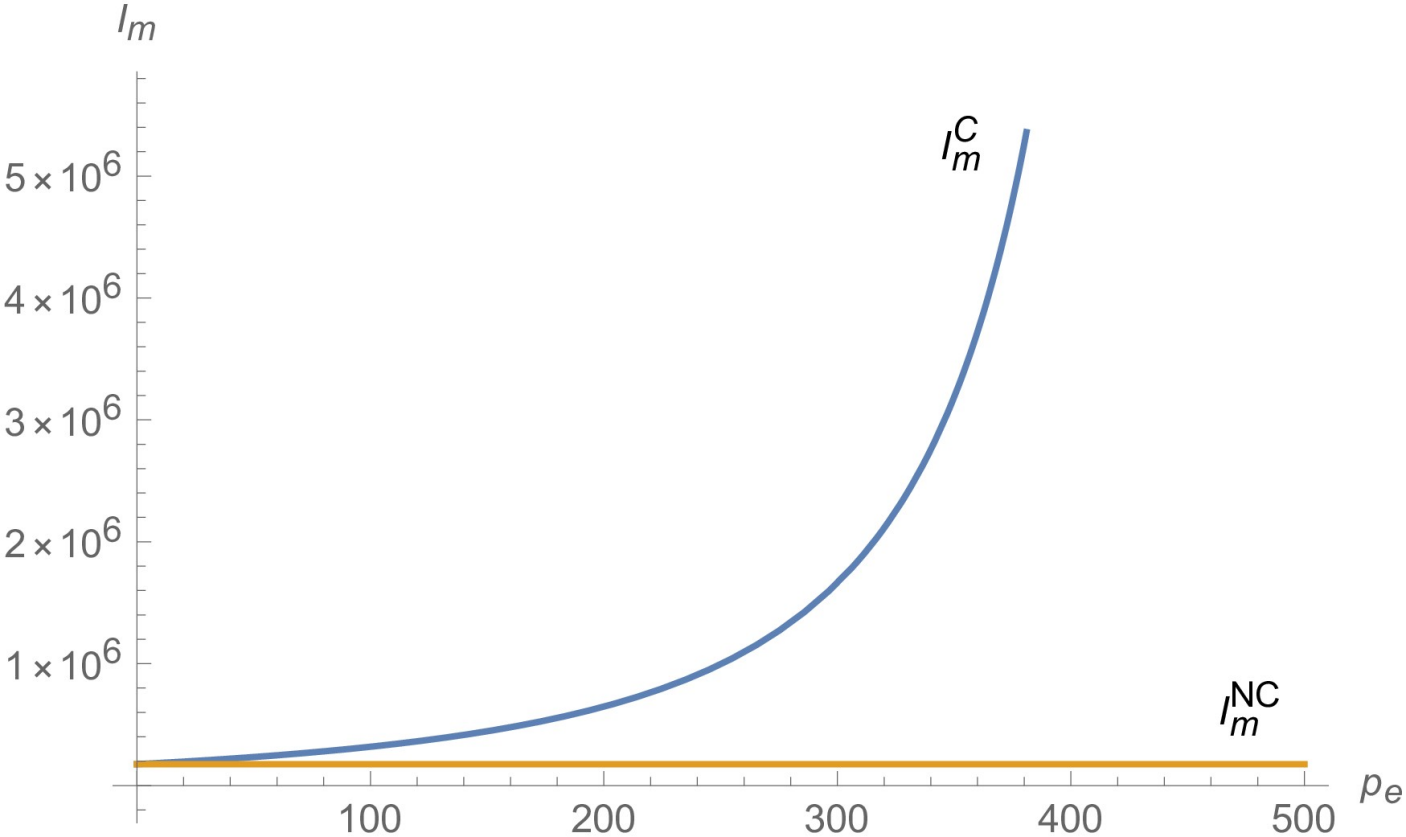
The impact of *p*_*e*_ on $I_{m}^{C}$.

**Fig 22:**
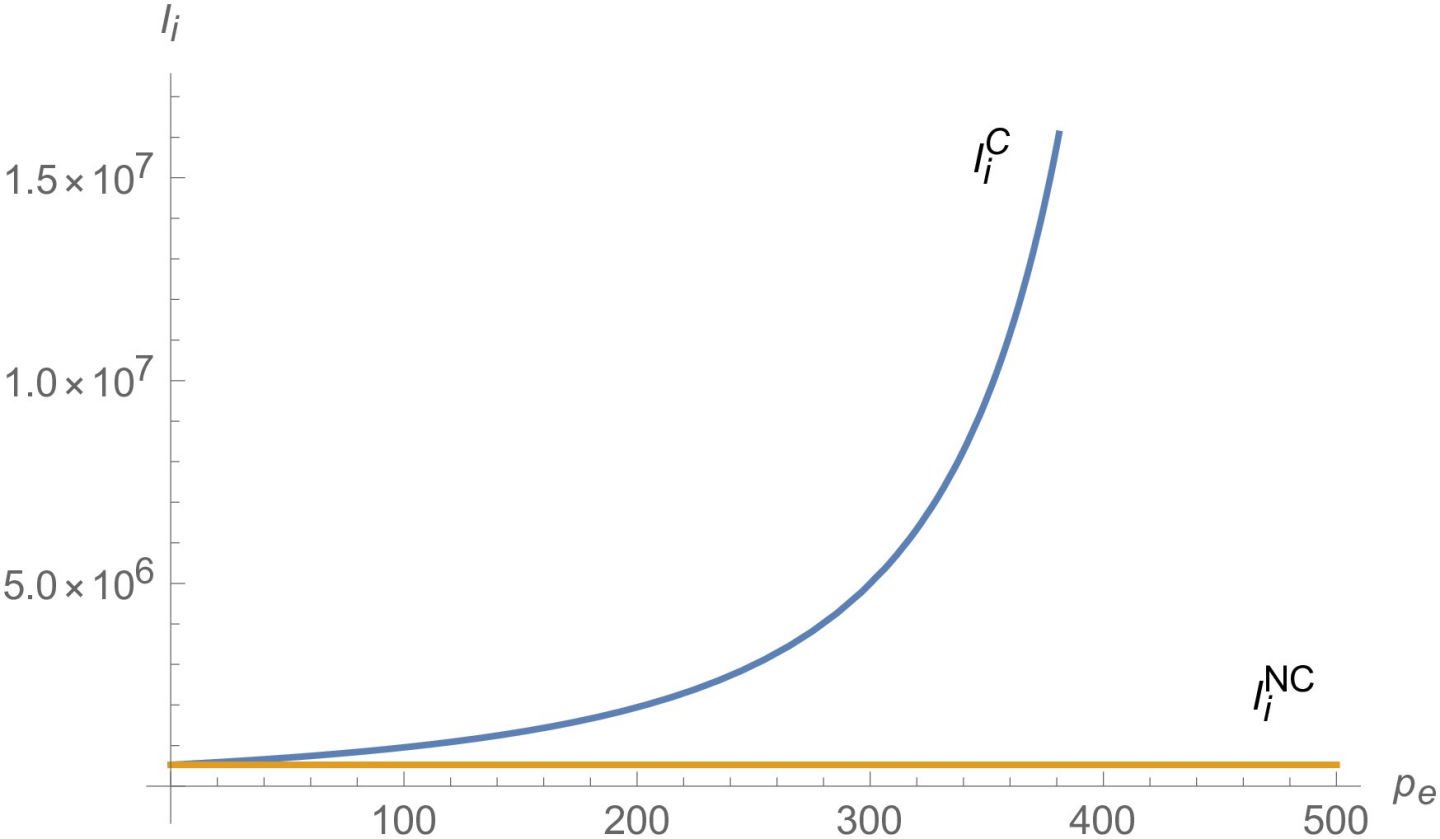
The impact of *p*_*e*_ on $I_{i}^{C}$.

**Fig 23:**
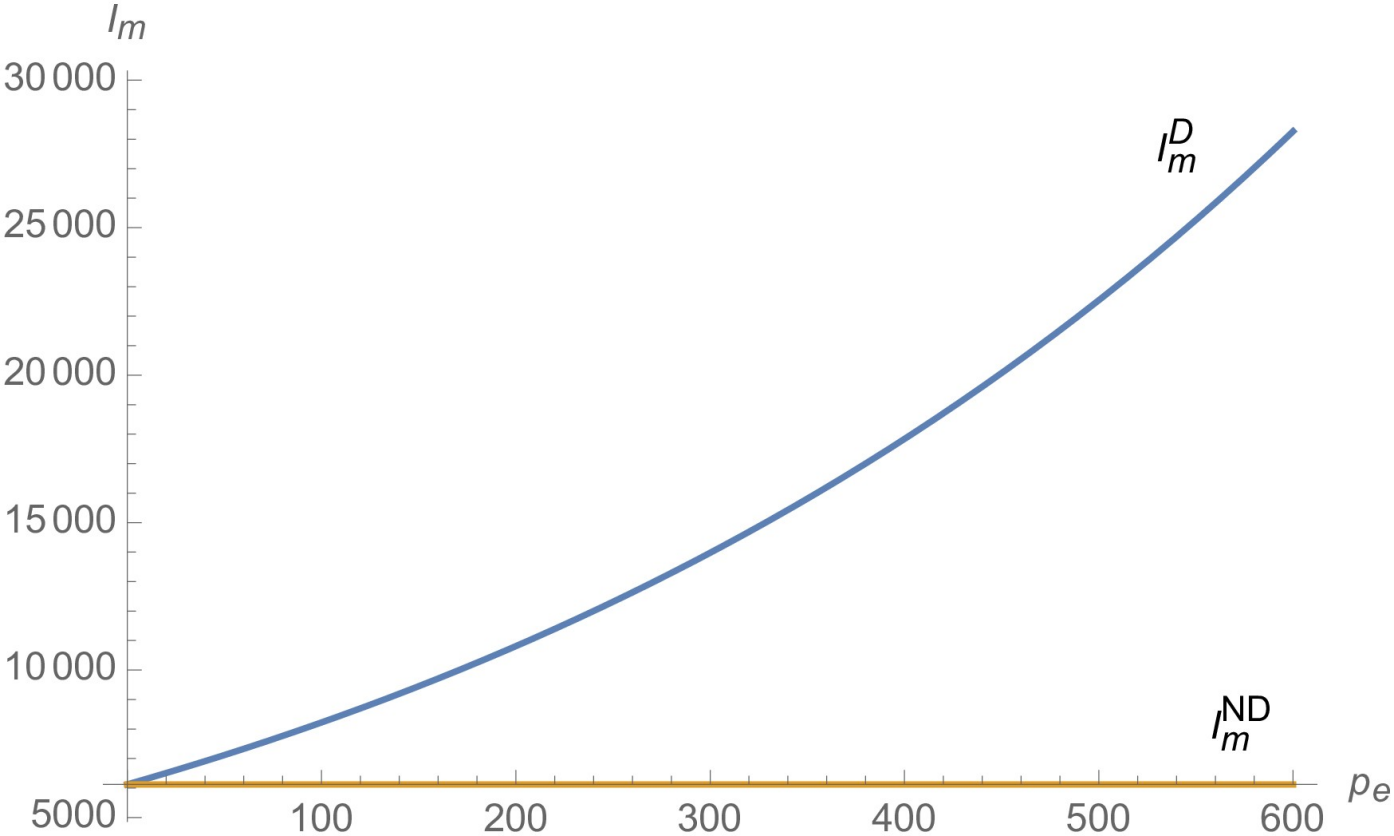
The impact of *p*_*e*_ on $I_{m}^{D}$.

**Fig 24:**
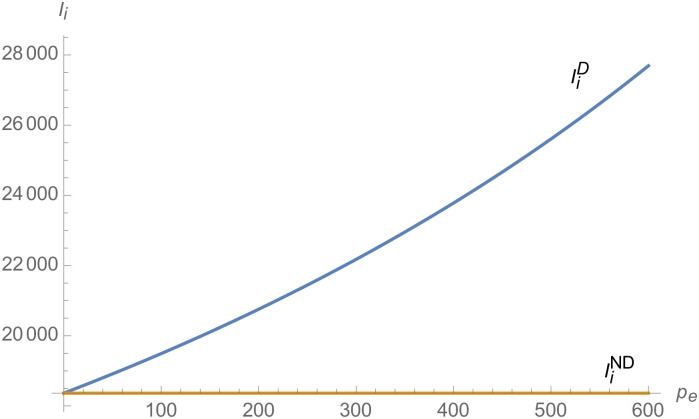
The impact of *p*_*e*_ on $I_{i}^{D}$.

From Figs 13–16, compared with no dual-credit policy, the implementation of dual-credit policy improves the technical level of NEVs, no matter which decision-making mode is adopted in the NEVs supply chain. From Figs 17 and 18, the output of NEVs under both decentralized and centralized decisions increases after the implementation of dual-credit policy. From Figs 19 and 20, the profit of NEVs supply chain increases, after the implementation of dual-credit policy. From Figs 21–24, the R&D investment of manufacturer and suppliers increases, after the implementation of dual-credit policy. In addition, no matter which decision-making mode is adopted in the supply chain, with the increase of the credit transaction price, the profit of NEVs supply chain will increase, the R&D investment of manufacturer and suppliers will increase, and the output and technical level of NEVs will increase.

Therefore, the dual-credit policy promotes the improvement of NEV technology level, realizes the scale growth of NEVs, stimulates manufacturer and suppliers to increase R&D investment, and improves the profit of NEVs supply chain.

#### The impact of negotiating power

**Fig 25:**
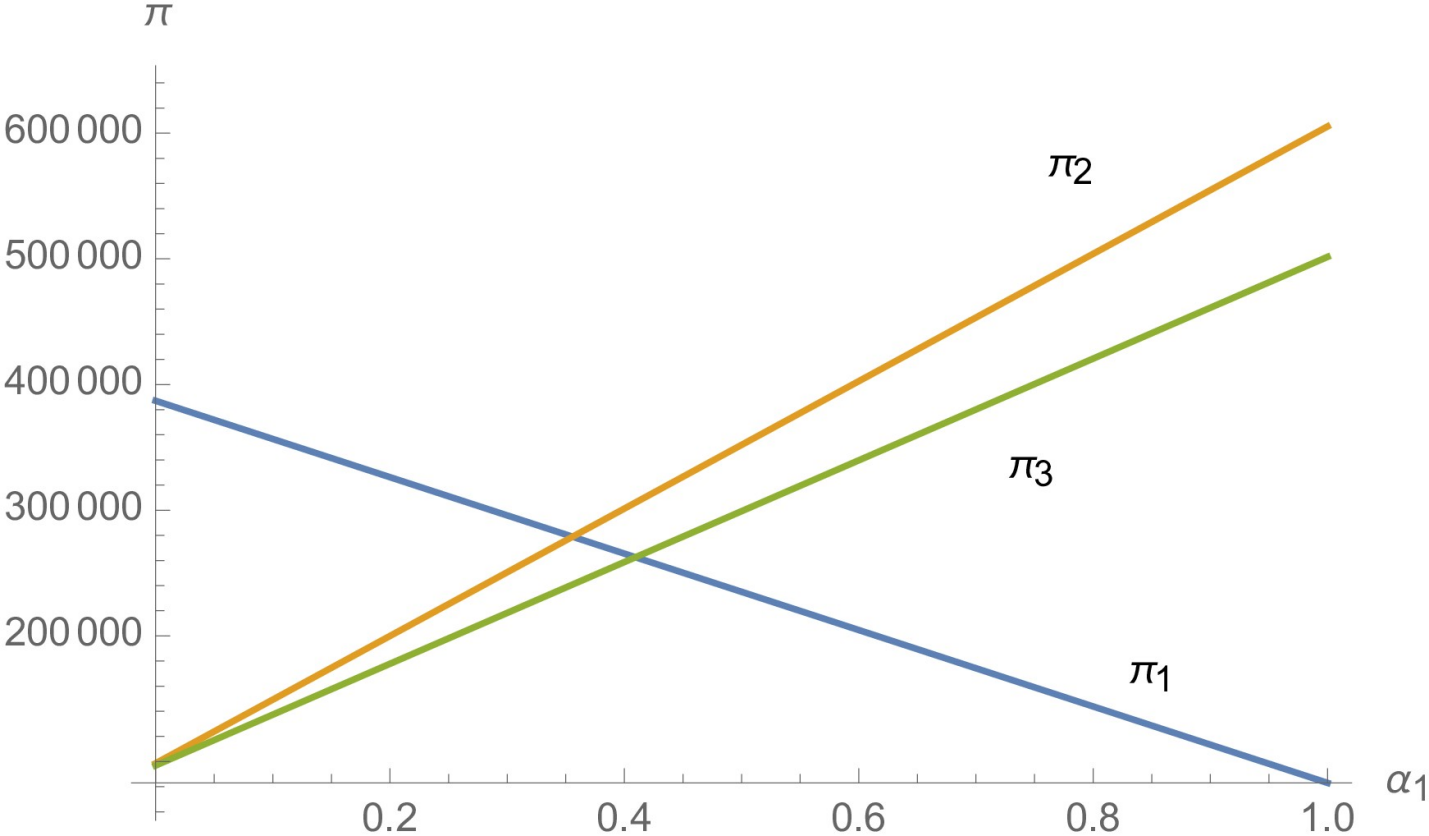
The impact of *α*_1_ on *π*.

**Fig 26:**
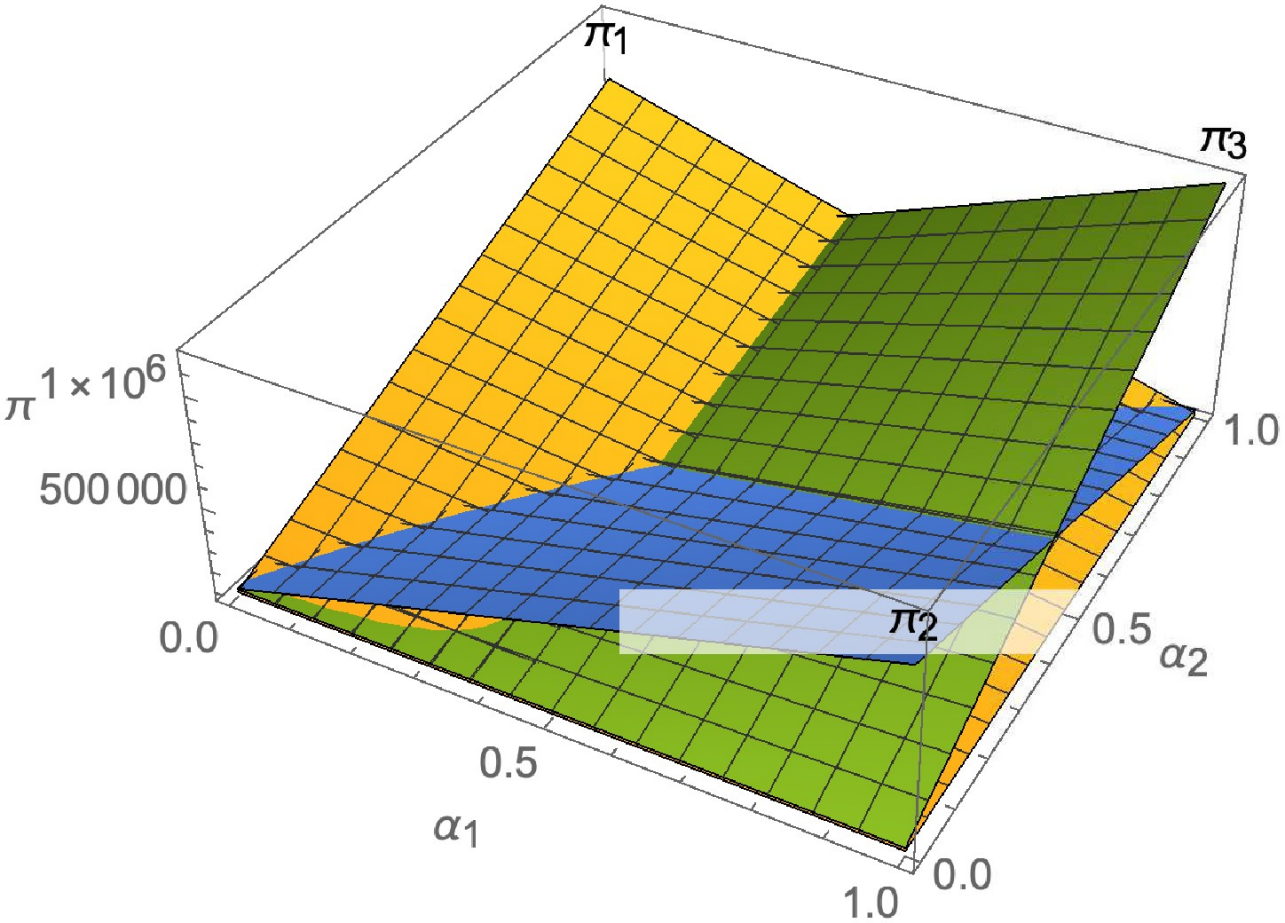
The impact of *α*_1_ and *α*_2_ on *π*_1_.

**Fig 27:**
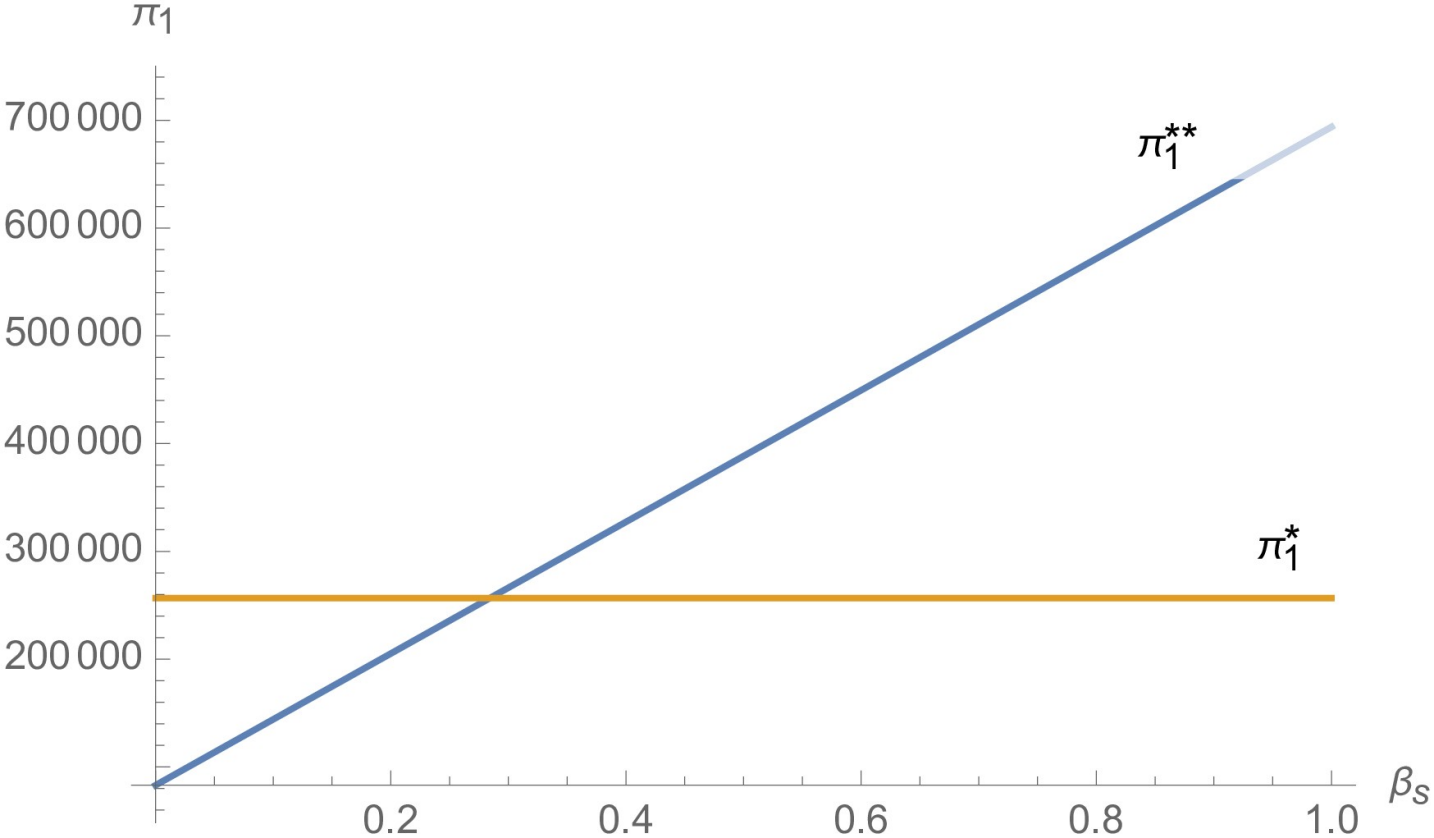
The impact of *β*_*s*_ on *π*_1_.

**Fig 28:**
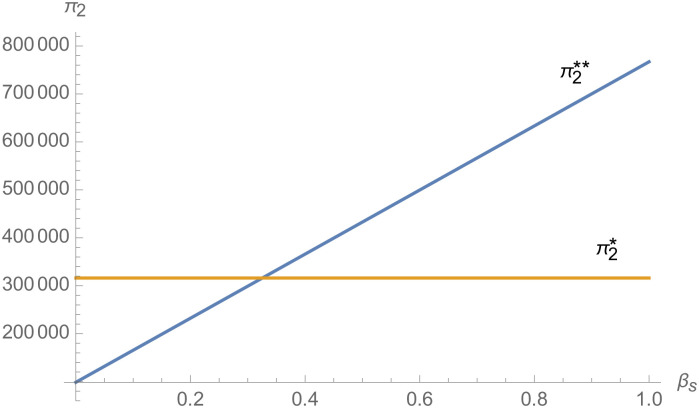
The impact of *β*_*s*_ on *π*_2_.

**Fig 29:**
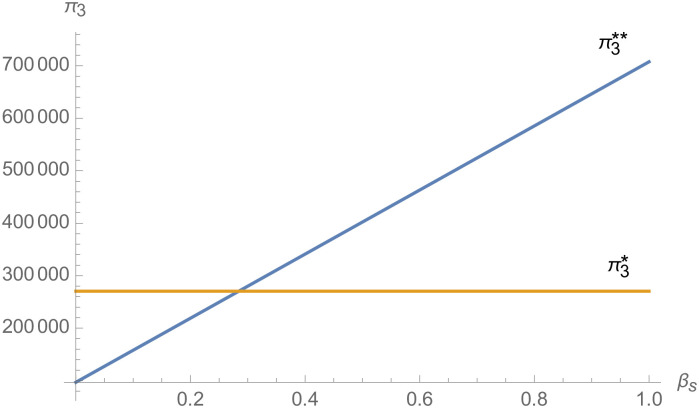
The impact of *β*_*s*_ on *π*_3_.

From Fig 25, with the improvement of manufacturer’s negotiating power *α*_1_ to supplier 1, the profit of supplier 1 decreases. However, with the improvement of manufacturer’s negotiating power *α*_1_ to supplier 1, the profits of suppliers 2 and 3 increase. From Fig 26, the profit of supplier 1 increases with the improvement of manufacturer’s negotiation power *α*_2_ and *α*_3_ with supplier 2 and supplier 3. From Figs 27–29, that whether suppliers form alliances depend on the negotiating power with the alliance, the stronger the negotiating power of the supplier alliance, the more profits the supplier will get. When the negotiating power of the alliance is low, the profit of the alliance negotiation is lower than that of the independent negotiation, and the suppliers will choose to negotiate independently. Suppliers will choose to form alliances and participate in negotiations only when the alliance negotiating power is high and the profit obtained by all suppliers through alliance negotiation is guaranteed to be higher than that of independent negotiation.

#### The impact of supplier number on NEVs supply chain

In the numerical analysis, we take the NEV supply chain composed of 3 suppliers and 1 manufacturer as an example. Further, we discuss the impact of the change in the number of suppliers *n* on the NEVs supply chain.

To intuitively show the impact of the number of suppliers on the NEV supply chain, we set *k*_*m*_ = *k*_1_ = *k*_2_ = ⋯ = *k*_*n*_ = 50, the negotiating power of suppliers *α*_1_ = *α*_2_ = ⋯ = *α*_*n*_ = 0.6, and the other parameters remain unchanged.

**Fig 30:**
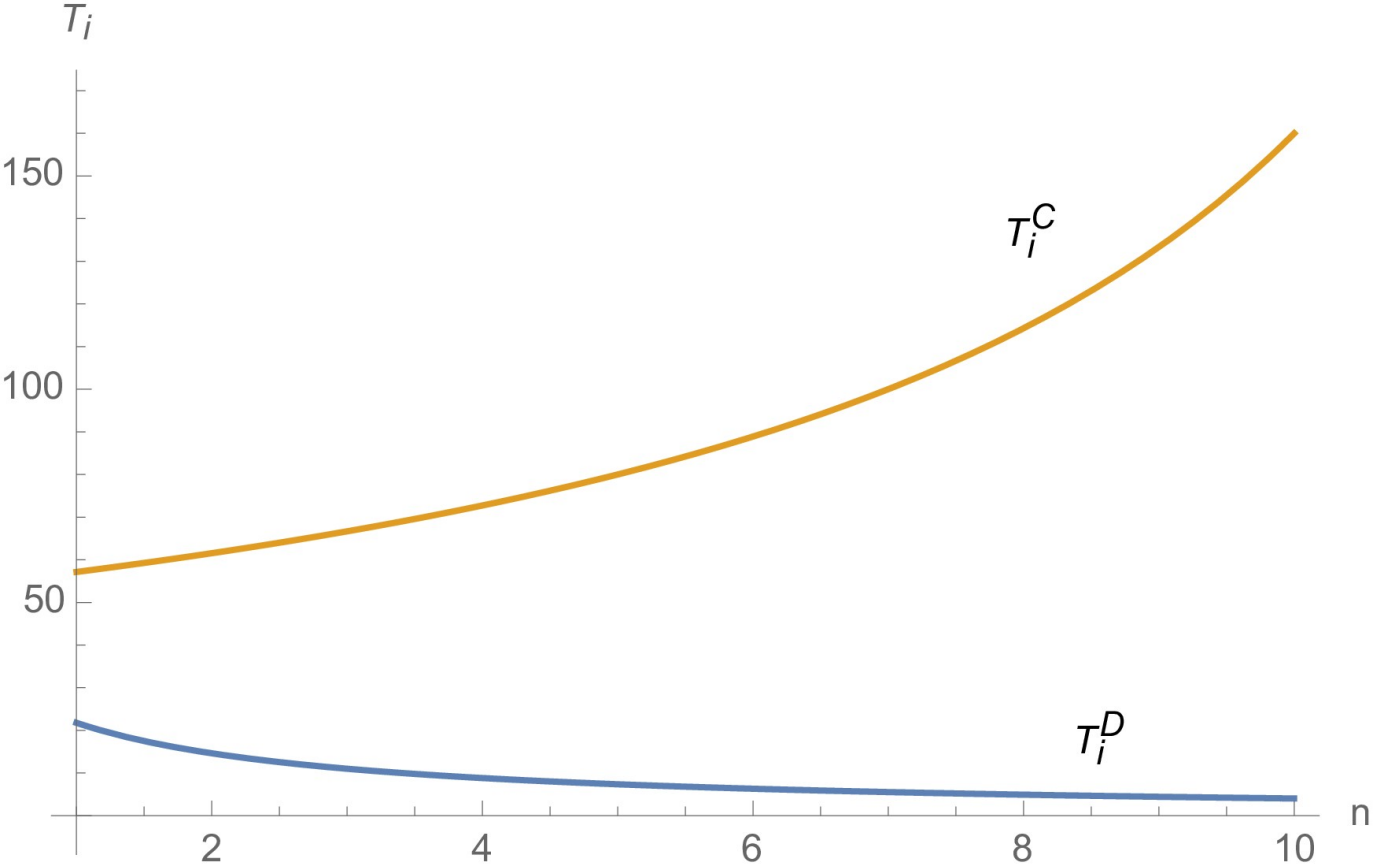
The impact of *n* on *T*_*i*_.

**Fig 31:**
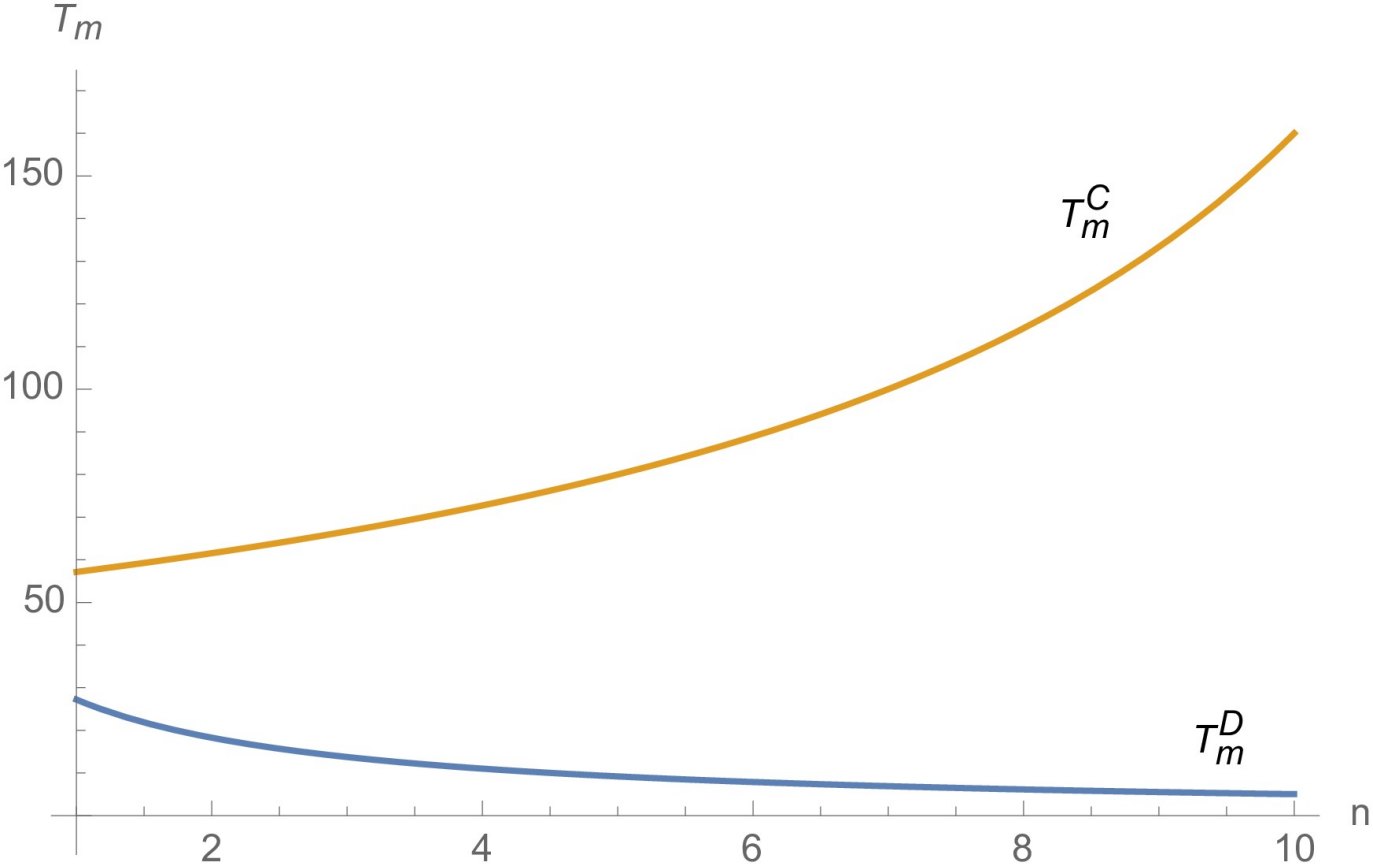
The impact of *n* on *T*_*m*_.

**Fig 32:**
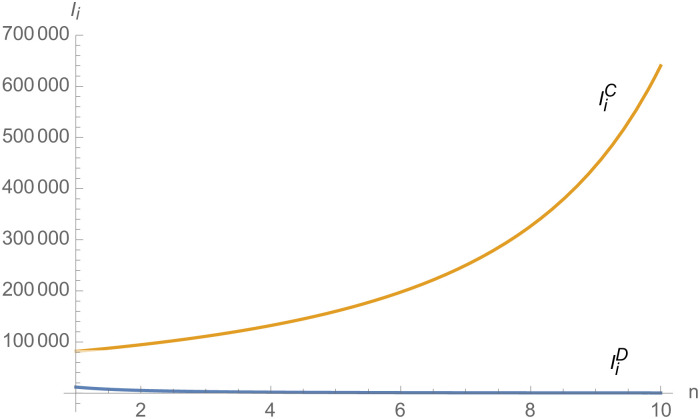
The impact of *n* on *I*_*i*_.

**Fig 33:**
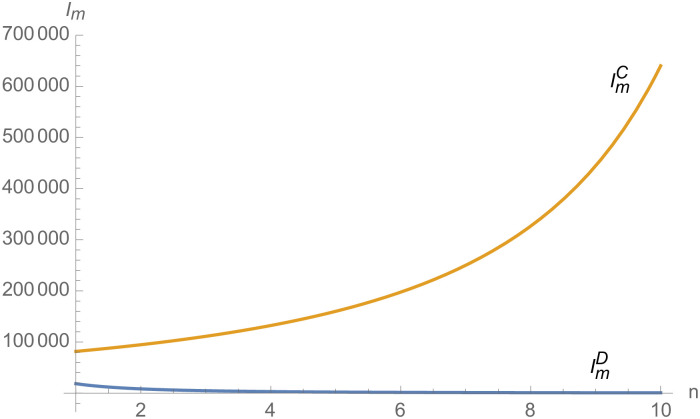
The impact of *n* on *I*_*m*_.

**Fig 34:**
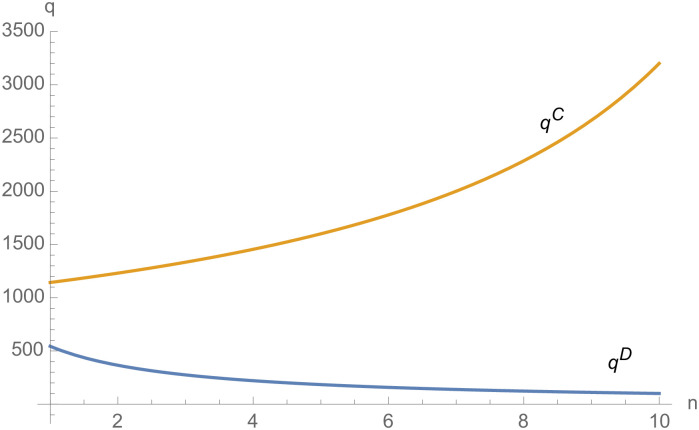
The impact of *n* on *q*.

**Fig 35:**
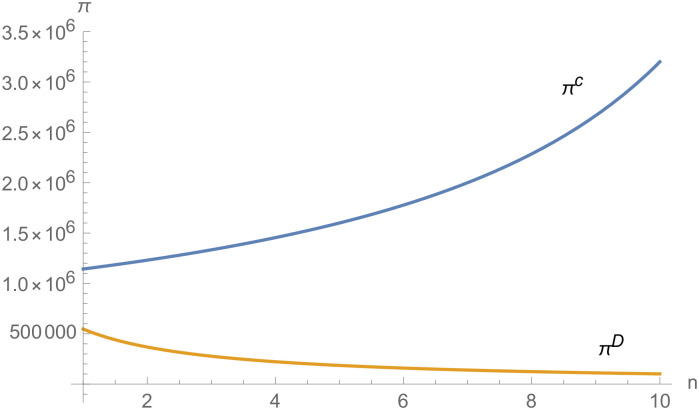
The impact of *n* on *π*.

**Fig 36:**
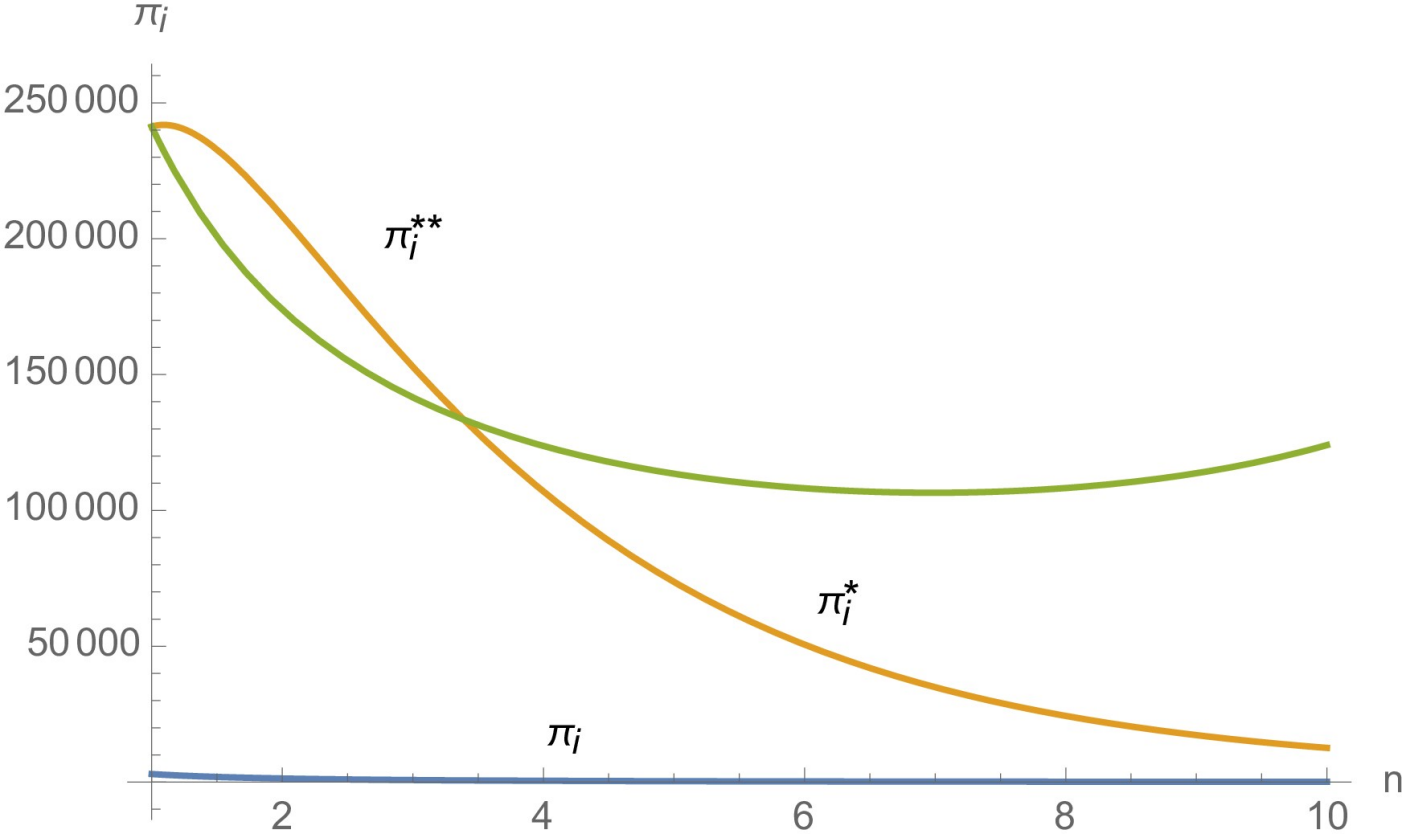
The impact of *n* on *π*_*i*_.

**Fig 37:**
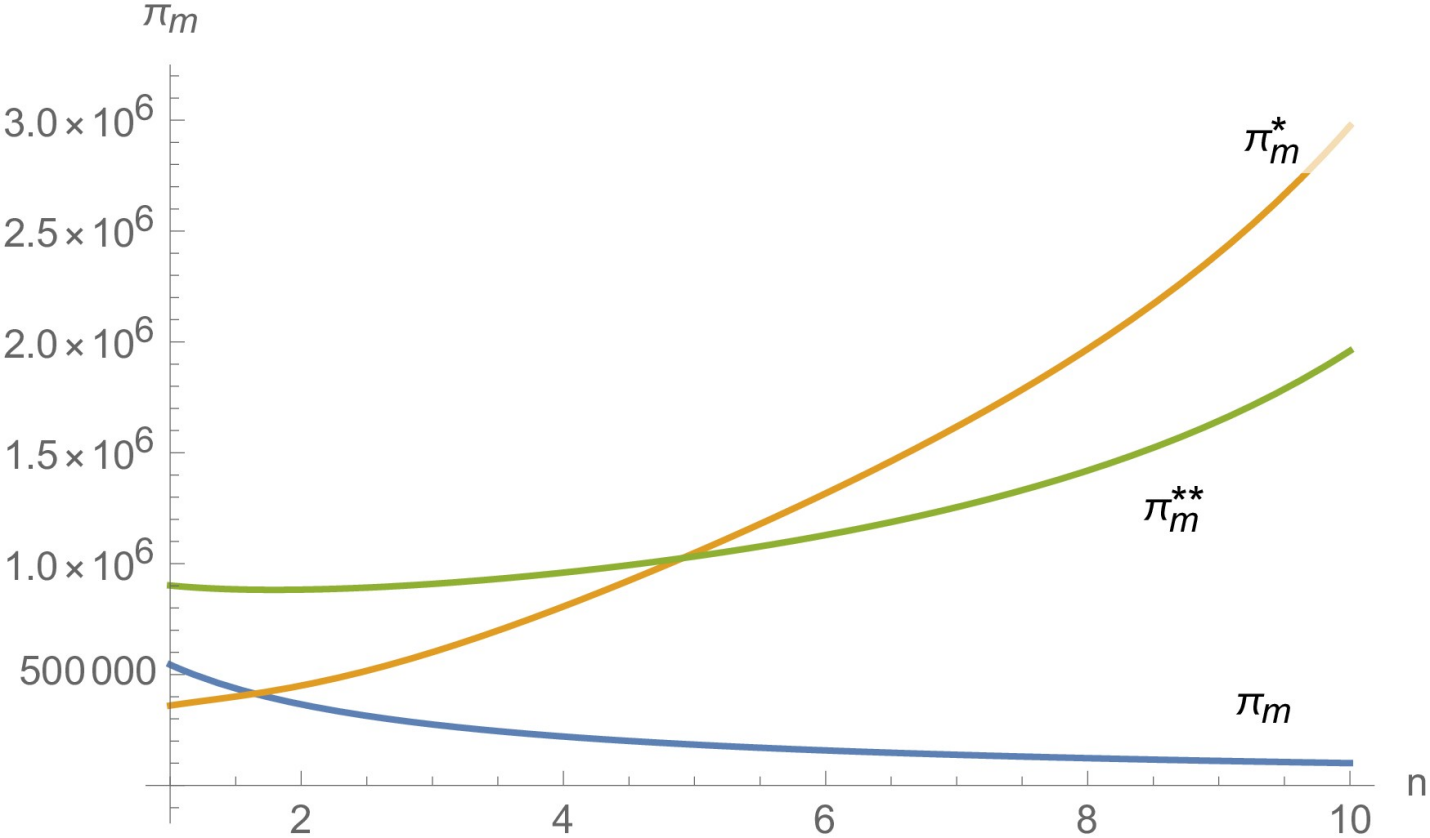
The impact of *n* on *π*_*m*_.

**Fig 38:**
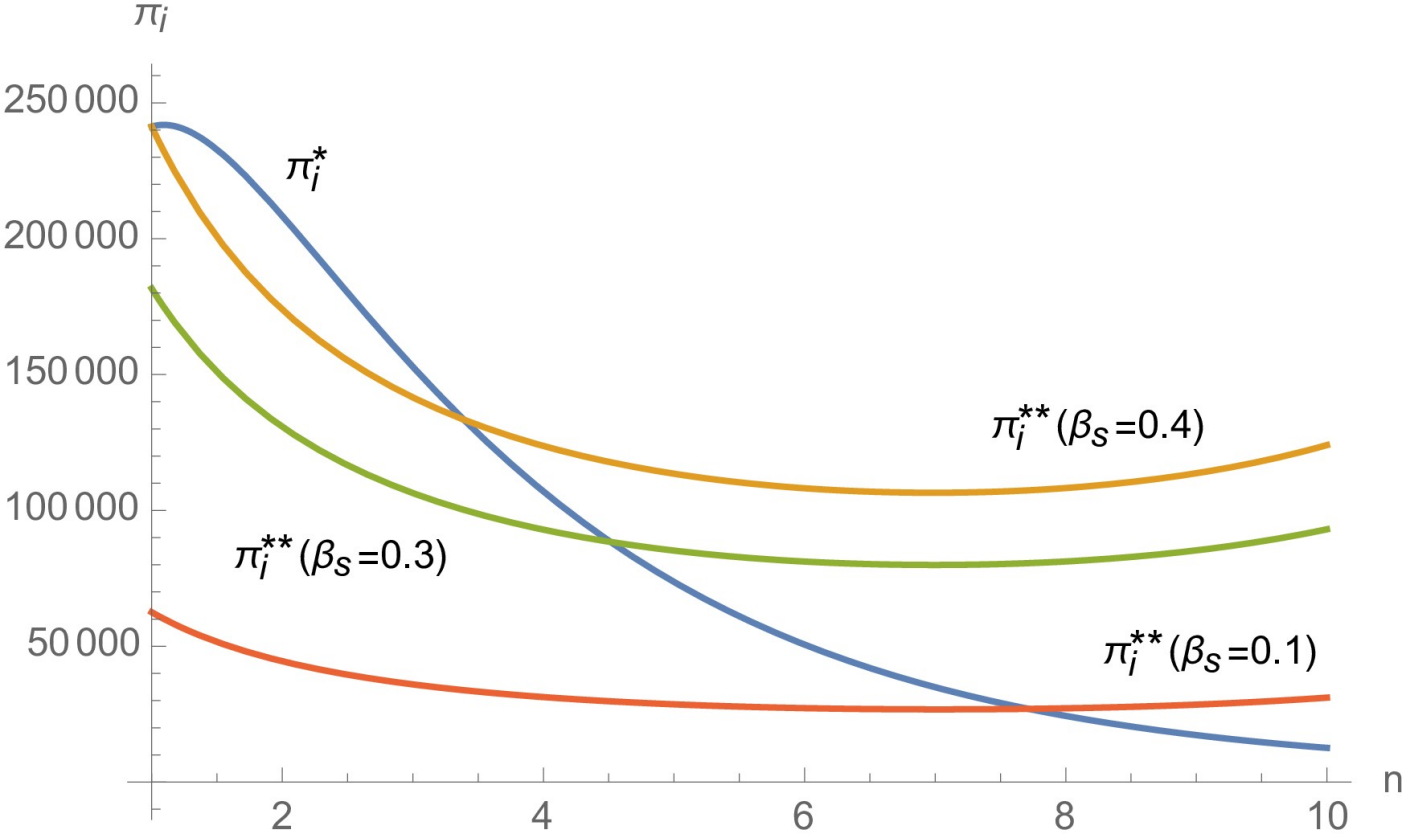
The impact of *n* on *π*_*i*_.

From Figs 30––35, with the increase of the number of suppliers, compared with decentralized decision-making, NEVs’ technical level, R&D investment, output, and supply chain profit are all increased under centralized decision-making, and the supply chain is more inclined to choose centralized decision-making. From Figs 36 and 37, when the profit distribution strategy of bargaining game is adopted in the supply chain, the profit of both suppliers and manufacturer is improved compared with decentralized decision, no matter whether the supplier negotiates independently or in alliance negotiation. From Fig 38, the stronger the negotiating power of the supplier alliance, the higher the profit of the supplier. However, suppliers do not always choose alliance. For example, when the number of suppliers is 5 and the negotiating power of the alliance is 0.1, suppliers will not choose alliance. Only when the negotiating power of the alliance is greater than 0.3, suppliers will choose alliance. However, with the increase of the number of suppliers, the alliance negotiating power of suppliers is getting lower and lower. When the number of suppliers is 4, the negotiating power of the alliance is greater than 0.4, and the suppliers will form an alliance. When the number of suppliers is 5 and the negotiating power of alliance is greater than 0.3, suppliers will consider alliance; When the number of suppliers is 10, the negotiating power of the alliance is greater than 0.1, the suppliers will consider the alliance.

## Conclusions

This paper assumes that the NEVs supply chain consists of a manufacturer and N parts suppliers, and the manufacturer purchases complementary parts from N parts suppliers. Under the dual-credit policy, the R&D investment of manufacturer and suppliers will affect the market demand and NEVs credit, we construct decentralized and centralized decision-making models under the dual-credit policy to obtain the optimal R&D investment strategy of the NEVs supply chain, and we make a comparative analysis of the optimal strategy under the two decision modes. Furthermore, considering that suppliers can form a supplier alliance to cooperate with manufacturer, we construct a bargaining game model under the condition of the non-alliance and alliance of suppliers, and discuss the coordination strategy of the supply chain, which provides a theoretical reference for supply chain coordination. Finally, a numerical example is given to verify the effectiveness of the coordination strategy.

It is found that under the dual-credit policy, the higher the credit coefficient of technology improvement, the higher the credit transaction price, and the higher the R&D investment of NEV supply chain. The dual-credit policy can effectively encourage NEVs supply chain to increase R&D investment, improve the technical level and output of NEVs, and profit of the supply chain, and promote the development of NEVs industry. Under the dual-credit policy, the bargaining model is adopted to distribute the incremental profit reasonably under the centralized decision, and the coordination of NEVs supply chain is realized. Compared to decentralized decision-making, supply chain R&D investment and profit are increased and the win-win situation for NEVs supply chain enterprises is realized. Suppliers of non-aligned negotiate with manufacturers, and the negotiation sequence and negotiating power will affect the profits of suppliers. Suppliers can get more profit by bringing the negotiation sequence forward. For lacking the right to determine the negotiation sequence, the suppliers will have to transfer the profits to the manufacturer to compete for the negotiations sequence, resulting in the suppliers can only be able to get the profits of the last round of the negotiations, which is the lowest profit that the supplier can get from the negotiations. Additionally, the manufacturer gets excess profit because it has the right to determine the negotiations sequence. However, suppliers can solve this problem by forming an alliance, reducing the profit loss of competing for the negotiation sequence, and increasing the profit of all suppliers when the alliance negotiating power reaches a certain threshold.

In addition, the negotiating power of suppliers will affect the price of parts and profit. In the case of supplier non-alliance, the stronger the negotiating power of the manufacturer to the supplier, the lower the parts price of the supplier and the lower the profit, while the higher the parts price of other suppliers, the higher the profit. In the case of a supplier alliance, the stronger the alliance negotiating power, the higher the parts price of suppliers, and the higher the profit.

Policy recommendations: (1) The dual-credit policy can promote the R&D investment of NEVs, improve the technical level and output of NEVs, and achieve technological breakthrough and industrial cultivation. (2) The policy factors will affect the R&D decision of NEV supply chain. The higher the credit coefficient of technology and the credit transaction price, the higher the R&D investment of supply chain, and the higher the output and technology level of NEVs. Therefore, the government can realize the regulation of NEVs industry and guide the healthy and sustainable development of NEVs industry by adjusting the credit coefficient of technology improvement or guiding the transaction price of credit. (3) NEVs supply chain centralized decision-making has more advantages, the government should encourage centralized decision-making among supply chain enterprises, which will be conducive to improving the technical level of NEVs and opening a new situation of NEV industry development.
